# In Vitro and In Silico Evaluation of the Antitumor Potential of New Benzotriazole–1,2,4-Triazole Hybrids

**DOI:** 10.3390/ph19101591

**Published:** 2026-10-08

**Authors:** Gabriel Mardale, Victor Dumitrașcu, Raluca Pop, Catalin Dumitru, Delia Muntean, Marius Mioc, Alexandra Mioc, Bogdan-Ionuț Mara, Mihaela Balan-Porcarasu, Oana Budu, Roxana Negrea-Ghiulai, Codruța Șoica

**Affiliations:** 1 Faculty of Pharmacy, “Victor Babes” University of Medicine and Pharmacy, Eftimie Murgu Square, No. 2, 300041 Timisoara, Romania; 2 Research Center for Experimental Pharmacology and Drug Design (X-Pharm Design), “Victor Babes” University of Medicine and Pharmacy, Eftimie Murgu Square, No. 2, 300041 Timisoara, Romania; 3 Faculty of Medicine, “Victor Babes” University of Medicine and Pharmacy, Eftimie Murgu Square, No. 2, 300041 Timisoara, Romania; 4 Coriolan Dragulescu Institute of Chemistry, Romanian Academy, Bv. M. Viteazu, No. 24, 300223 Timisoara, Romania; 5 Petru Poni Institute of Macromolecular Chemistry, 41A Aleea Gr. Ghica Vodă, 700487 Iaşi, Romania

**Keywords:** benzotriazole hybrids, 1,2,4-triazole, heterocyclic compounds, anticancer activity, molecular docking, molecular dynamics

## Abstract

**Background/Objectives**: One of the major problems facing the medical world is cancer, which causes millions of deaths each year. Nitrogen-containing heterocycles appear to offer a potential solution to this problem. Thus, the present study aimed to synthesize and characterize—both physicochemically and biologically—the new compounds obtained by combining benzotriazole and 1,2,4-triazole scaffolds. **Methods**: Each of the four cell lines (HaCaT—human keratinocyte, A375—melanoma, A549—non-small cell lung cancer, and HT-29—colorectal adenocarcinoma) was treated for 48 h with concentrations ranging from 10 to 100 µM of the four compounds. After 48 h of treatment, changes in cell viability were assessed using the Alamar Blue assay. These findings were supplemented by microscopic analysis of metabolic changes, as well as alterations in the actin cytoskeleton and nuclei, using staining techniques. **Results**: The results showed that **MG-1** (p-chlorophenyl-) and **MG-3** (p-(N,N-dimethylamino)phenyl-) are the most active compounds in our study, with IC_50_ values for each tumor line as follows: 75.43 ± 2.77, 77.15 ± 1.32, and 96.28 ± 1.72 μM in A375, A549, and HT-29 cells, and 78.97 ± 3.08, 56.78 ± 2.57, and 69.19 ± 3.97 μM, respectively. In HaCaT cells, IC_50_ values remained above 100 μM for all four hybrids. In silico screening identified MEK1 as the most favorable target for the investigated series, while 100 ns molecular dynamics simulations and MM-GBSA analysis supported stable binding of **MG-1** and **MG-3**, with predicted binding free energies of −54.83 and −40.70 kcal/mol, respectively. **Conclusions**: Even with a limited set of compounds, we can conclude that the biological effect observed in our study depended on both the cell line and the substituent attached to the aryl moiety. The results obtained for **MG-1** and **MG-3** suggest that these compounds may serve as suitable starting points for further structural optimization and investigation of the mechanisms of action.

## 1. Introduction

**Figure 1:**
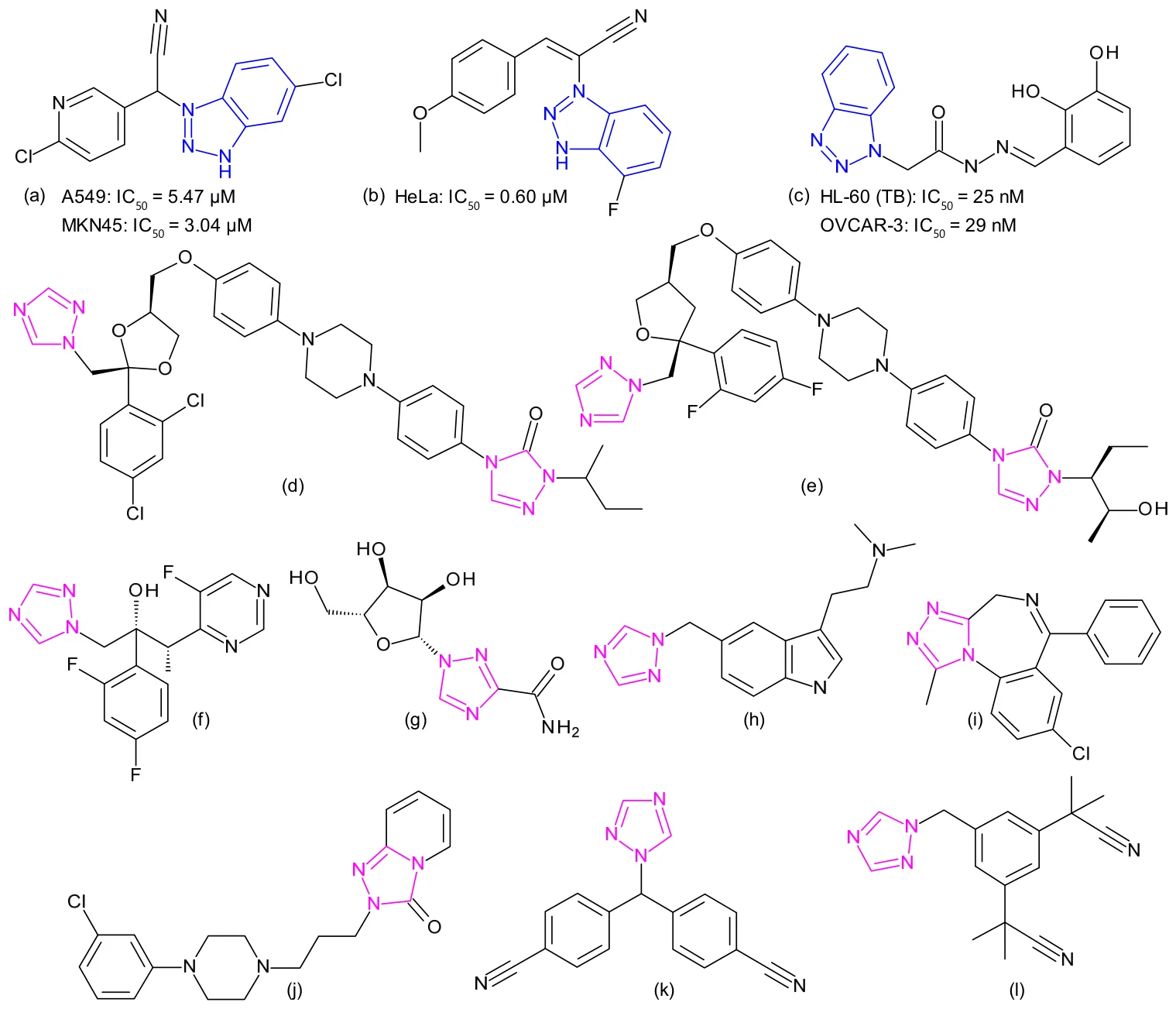
Chemical structures of representative bioactive benzotriazole hybrids and selected 1,2,4-triazole-containing drugs discussed in the Introduction. Benzotriazole scaffolds in compounds (**a**–**c**) are highlighted in blue, while 1,2,4-triazole rings in compounds (**d**–**l**) are highlighted in magenta. Compounds (**a**–**c**) correspond to the previously reported benzotriazole derivatives by Li et al. [8], Riu et al. [9], and Kassab et al. [10], respectively; their reported antitumor activity is indicated below each structure. Compounds (**d**–**l**) are itraconazole, posaconazole, voriconazole, ribavirin, rizatriptan, alprazolam, trazodone, letrozole, and anastrozole, respectively.

Cancer is the leading cause of mortality and morbidity worldwide, and the identification of new, more selective and more potent therapeutic agents remains the primary goal of biomedical research [1]. In this context, the development of small molecules with relevant antitumor potential represents both a major area of interest and a significant challenge for research in the field of medicinal chemistry [2]. Thus, in order to identify such therapeutic agents, researchers have turned their attention to nitrogen-containing heterocycles where the benzotriazole ring has emerged as an interesting scaffold. Benzotriazole is able to play a main role in the synthetic development of other compounds due to its structural flexibility that allows the introduction of various substituents via straightforward chemical reactions, leading to new compounds. Several benzotriazole derivatives have been associated with a very large therapeutic spectrum of biological activities, including antimicrobial [3], antiviral [4], anti-inflammatory [5], and antitumor [6,7]. In oncology, the scientific literature refers to specific benzotriazole derivatives with significant antitumor activity, as revealed by their strong inhibitory effects on cell viability, cell cycle arrest, pro-apoptotic activity or the ability to target specific molecules relevant to tumor progression. For example, the benzotriazole derivatives synthesized by Li et al. were tested on several tumor lines (VX2, A549, MKN45, and MGC). According to the study, the compound 2-(5-chloro-1*H*-benzo[d][1,2,3]triazol-1-yl)-3-(6-chloropyridin-3-yl)acrylonitrile (Figure 1a) most effectively inhibited cell growth in the A549 (IC_50_ = 5.47 µM) and MKN45 (IC_50_ = 3.04 µM) [8]. Another derivative from the series by Riu et al., 2-(4-fluoro-1*H*-benzo[d][1,2,3]triazol-1-yl)-3-(4-methoxyphenyl) acrylonitrile (Figure 1b), arrested the cell cycle in the G_2_/M phase of the HeLa and MCF-7 tumor cell lines, demonstrating pro-apoptotic potential [9]. Compound 2-(1*H*-benzo[d][1,2,3]triazol-1-yl)-N′-(2,3-dihydroxybenzyl-idene)acetohydrazide (Figure 1c) from the series by Kassab et al. demonstrated the best effect on the HL-60 tumor line (IC_50_ = 25 nM), effectively inhibiting focal adhesion kinases (IC_50_ = 32.72 nM) and inducing elevated levels of caspase-3, consequently enhancing apoptosis [10].

Even though several reports have been published on the antitumor activity of benzotriazole derivatives, the biological insights persist limited [11]; thus, a recent study characterizing the preclinical results of benzotriazole in anticancer treatments indicates the existence of methodological heterogeneity that still hampers their clinical assessment. Additionally, the safety profile as well as the structure–activity relationships are not fully clarified for numerous compound series [12]. Thus, the synthesis and biological evaluation of a new series of benzotriazole hybrids remains highly justified, particularly in terms of their antitumor potential; additionally, the identification of structure–activity relationships could provide important information for the development of upcoming research in the field.

The choice of 1,2,4-triazol as hybridization partner for benzotriazole is justified by its dipolar character, ability to form hydrogen bonds, aromatic character, and water solubility which make it an important pharmacophore [13]. This particular scaffold can be found in numerous compounds currently used as drugs against various pathologies: antifungal (itraconazole [14], posaconazole [15], voriconazole [16]), antiviral (ribavirin [17]), antimigraine (rizatriptan [18]), anxiolytic (alprazolam [19]), antidepressants (trazodone [20]), and antitumor agents [21] (letrozole, anastrozole) (Figure 1).

Based on these considerations, our main objective was the synthesis of new benzotriazole–1,2,4-triazole hybrids with potential antitumor effect, followed by their physicochemical and biological evaluation.

## 2. Results

### 2.1. Chemistry

**Scheme 1:**
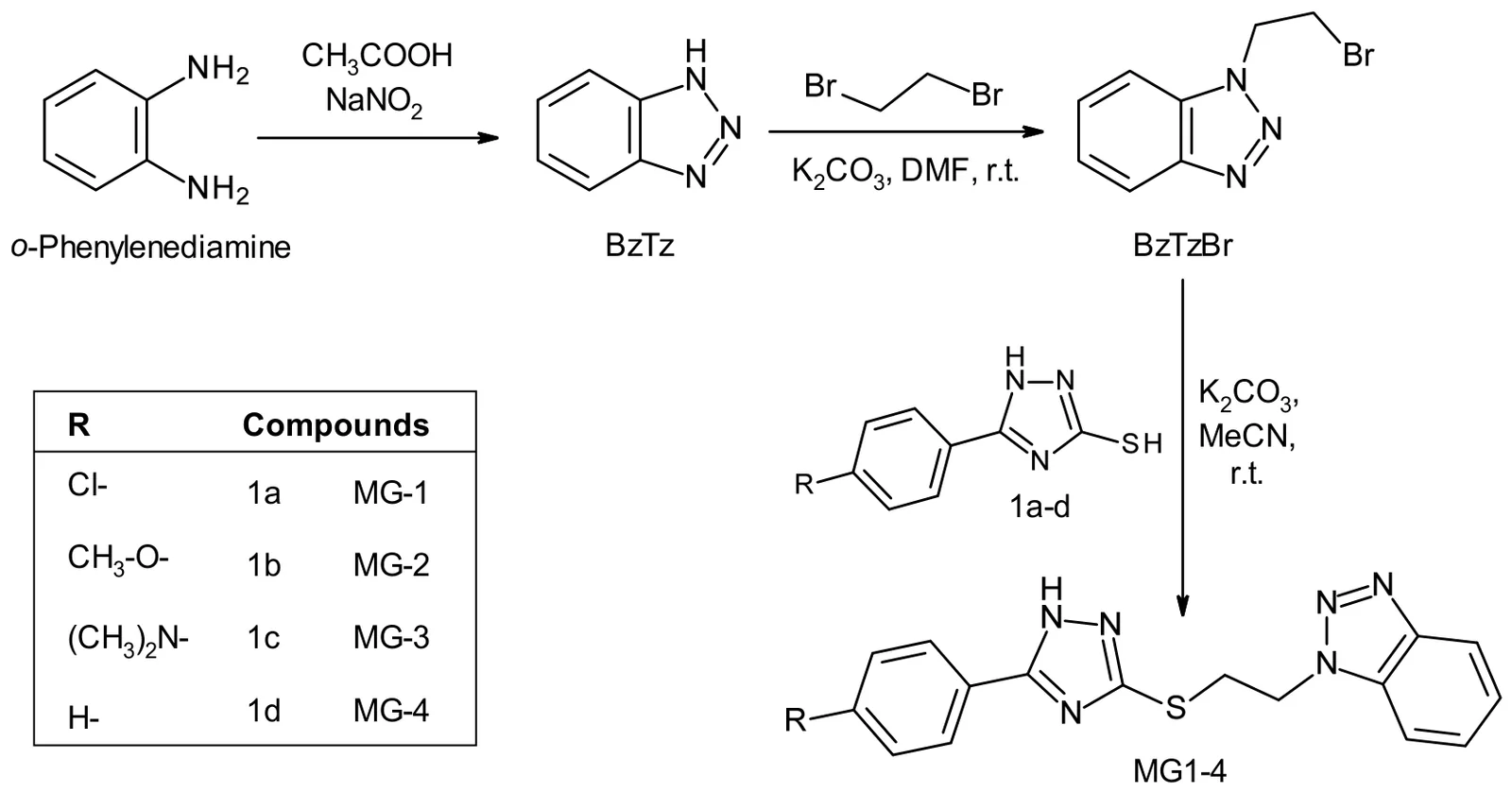
Synthesis of 1,2,4-triazol-benzotriazole derivatives; **MG-1** (1-[2-[[5-(4-chlorophenyl)-1*H*-1,2,4-triazol-3-yl]sulfanyl]ethyl]benzotriazole); **MG-2** (1-[2-[[5-(4-methoxyphenyl)-1*H*-1,2,4-triazol-3-yl]sulfanyl]ethyl]benzotriazole); **MG-3** (4-[3-[2-(benzotriazol-1-yl)ethylsulfanyl]-1*H*-1,2,4-triazol-5-yl]-N,N-dimethyl-aniline); **MG-4** (1-[2-[(5-phenyl-1*H*-1,2,4-triazol-3-yl)sulfanyl]ethyl]benzotriazole); BzTz (1*H*-benzotriazole); BzTzBr (1-(2-bromoethyl)-benzotriazole); DMF (Dimethylformamide); MeCN (Acetonitrile); r.t. (room temperature).

Compounds **MG-1**–**MG-4** were obtained by linking 1N from 1*H*-benzotriazole with the thiol group of compounds **1a**–**d** with the aid of 1,2-dibromoethane. Reaction pathways are depicted in Scheme 1. The compounds were obtained with various yields ranging from 21% to 51.4%. When analyzing the ^1^H and ^13^C NMR spectra of **MG-1**–**MG-4**, we observed that some of the peaks were doubled and/or broadened. This behavior was also observed in diacetylbetulin derivatives containing 5-substituted-1,2,4-triazoles at C30 [22] and was attributed to the existence of a slow exchange tautomeric equilibrium that can occur in 3,5-disubstituted 1,2,4-triazoles [23,24]. Adding TFA speeded up the tautomeric equilibrium leading to a fast exchange system and the NMR spectra show only one set of narrower peaks. The ^1^H NMR spectra show the protons from the phenyl and benzotriazole residues resonating between 8.3 and 7.0 ppm, the ethylenic protons at 5.1 and 3.7 ppm, protons of the methoxy group of **MG-2** at 3.8 ppm and the protons of the dimethylamino group of **MG-3** at 3.0 ppm. The ^13^C NMR spectra also confirm the proposed structures, showing the peaks at the appropriate chemical shifts (Figures S1, S2, S4, S5 and S7–S9).

### 2.2. General Biological Screening Strategy

The biological analysis focused on the final benzotriazole hybrids (**MG-1**–**MG-4**) and BzTz as the scaffold compound, and biological controls were used as reference standards for the analysis. Furthermore, the biological analysis was conducted under similar experimental conditions for the corresponding synthetic intermediates to demonstrate that the biological effects obtained are determined exclusively by the final compounds. Thus, the biological experimental data of the synthetic intermediates are found in Supplementary Material (Table S1 and Figures S14–S17); this means that the main discussion highlights the results obtained for BzTz and its final compounds. An additional antimicrobial screening of BzTz, the synthetic intermediates, and the final compounds was also performed; the corresponding methodology and results are provided in the Supplementary Materials (Tables S2 and S3).

### 2.3. The Effects of Benzotriazole and Its Hybrids on Cell Viability

HaCaT, A375, A549, and HT-29 were treated for 48 h with the benzotriazole parent compound (BzTz), the corresponding synthetic intermediates (**1a**–**d**), or the four final benzotriazole–1,2,4-triazole hybrids (**MG-1**–**MG-4**). After 48 h, the effects of the treatment were evaluated using the Alamar Blue assay, according to the protocol described in Section 4.2.2. The purpose of the assay was to detect and observe both metabolic changes and changes in cell viability, thereby assessing the effects of the studied compounds on cellular metabolic viability. The results obtained for the synthetic BzTz intermediates (**1a**–**d**) are presented in Supplementary Material (Table S1 and Figures S14–S17). The effect of BzTz at the highest concentration (100 μM) was used as a reference, in comparison with the effects generated by the compounds at all tested concentrations. The highest concentration was used for BzTz because the IC_50_ within the tested range could not be determined for it, and as a reference compound to assess whether derivatization results in stronger effects at lower or similar concentrations (Table 1).

**Figure 2:**
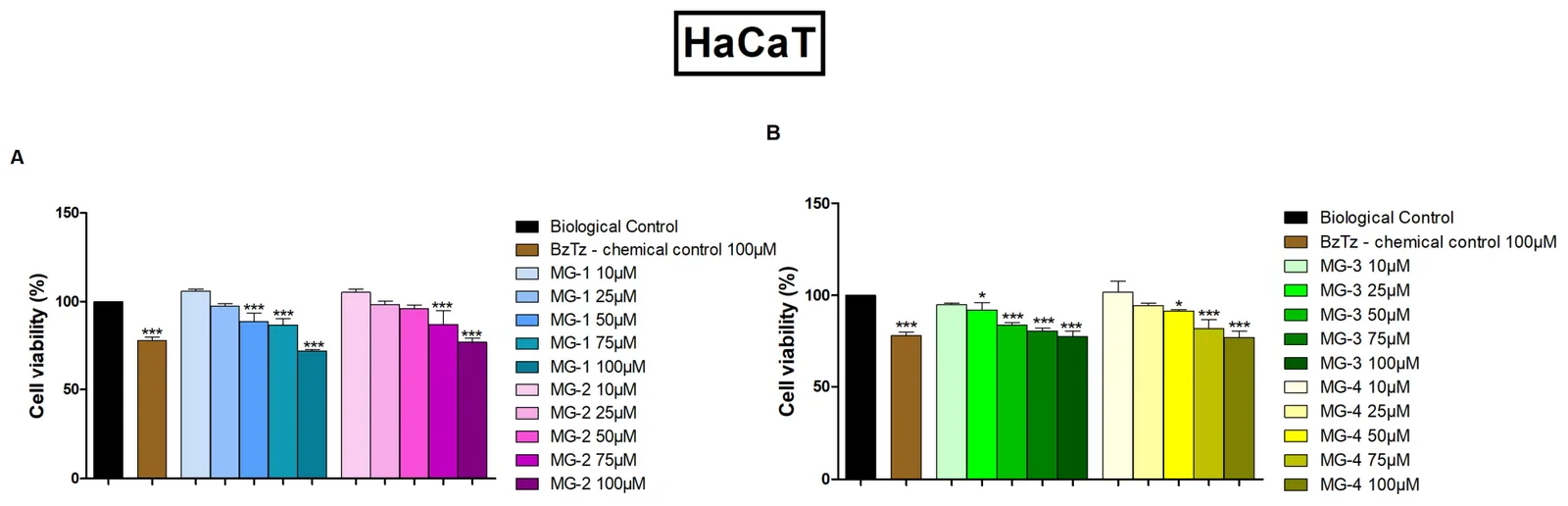
Viability of HaCaT cells after 48 h of treatment with compounds **MG-1**, **MG-2**, **MG-3**, and **MG-4** at concentrations of 10, 25, 50, 75, and 100 μM. (**A**) Cell viability following treatment with **MG-1** and **MG-2**; (**B**) Cell viability following treatment with **MG-3** and **MG-4**. BzTz at 100 μM was included as the reference condition for the parent scaffold. Cell viability was assessed using the Alamar Blue assay and expressed as percentage relative to the biological control (100%). Data represent mean ± SD of three independent experiments performed in triplicate. Differences were considered statistically significant at *p* < 0.05 (* *p* < 0.05, and *** *p* < 0.001).

After the treatment of HaCaT cell line, a limited reduction in cell viability was observed for the parent compound (BzTz) only at the highest tested concentration of 100 μM (78.06% ± 1.9) compared to the biological control—untreated cells (100%). For the **MG-1** compound, the reduction in cell viability started at 50 μM, where the cell survival rate was 88.71% ± 4.8, which decreased as the test concentration increased as follows: at 75 μM, it reached 86.56% ± 3.64, and at 100 μM, it was 72.01% ± 0.87. In contrast, **MG-2** showed a modest effect on viability at 75 μM (86.92% ± 7.08), with the effect becoming more pronounced at 100 μM, where cell viability decreased to 76.99% ± 2.4. **MG-3** reduced cell viability even at 25 μM (91.83% ± 4.09), then 83.83% ± 1.39 at 50 μM, 80.76% ± 1.56 at 75 μM, and 77.55% ± 3.2 at 100 μM. **MG-4** also showed a moderate viability-reducing effect at 50 μM (91.33% ± 0.99), becoming more evident at 75 μM (81.95% ± 4.85) and 100 μM (77.08% ± 3.68). There does not appear to be a significant difference between the effect of the parent compound (BzTz) and its hybrids (**MG-1**, **MG-2**, **MG-3**, **MG-4**) in this line, which means that derivatization did not result in any additional effect beyond that exhibited by benzotriazole (Figure 2).

**Figure 3:**
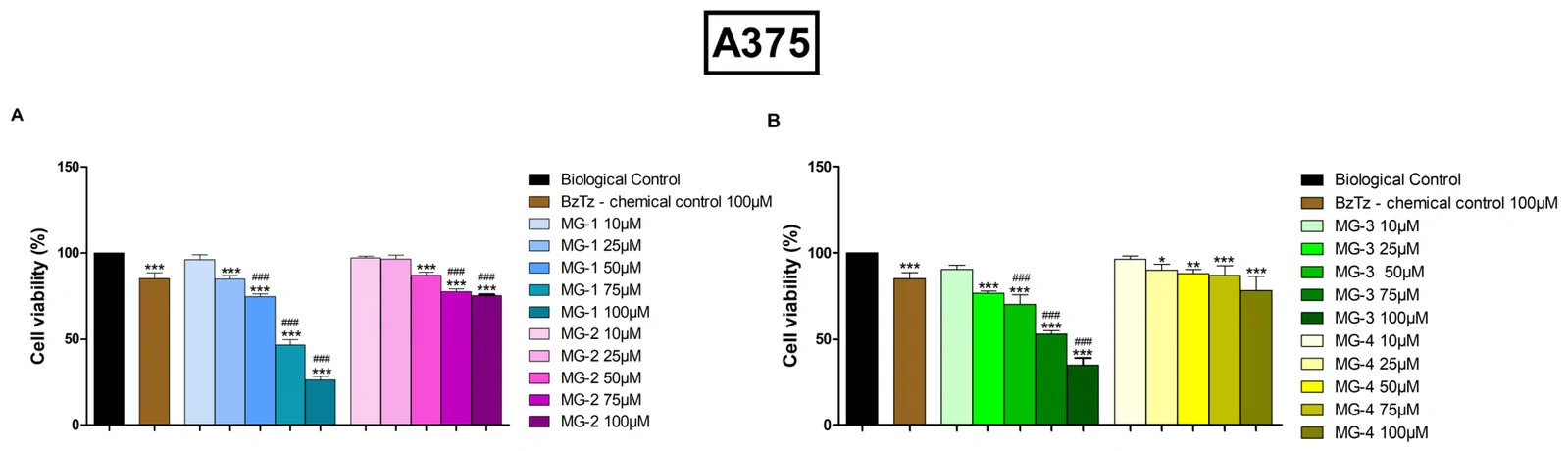
Viability of A375 cells after 48 h of treatment with compounds **MG-1**, **MG-2**, **MG-3**, and **MG-4** at concentrations of 10, 25, 50, 75, and 100 μM. (**A**) Cell viability following treatment with **MG-1** and **MG-2**; (**B**) Cell viability following treatment with **MG-3** and **MG-4**. BzTz at 100 μM was included as the reference condition for the parent scaffold. Cell viability was assessed using the Alamar Blue assay and ex-pressed as percentage relative to the biological control (100%). Data represent mean ± SD of three independent experiments performed in triplicate. Differences were considered statistically significant at *p* < 0.05 (* *p* < 0.05, ** *p* < 0.01 and *** *p* < 0.001). Hash symbols indicate statistically significant differences relative to benzotriazole tested at 100 μM, which was used as the reference condition for the parent scaffold because no IC_50_ value was reached within the tested concentration range, ### *p* < 0.001).

In the A375 melanoma cell line, the parent compound (BzTz) showed only a modest reduction in viability at the highest concentration (100 μM), where cell viability was 85.21% ± 3.35 compared to the biological control (100%). In contrast, **MG-1** showed a marked concentration-dependent reduction in cell viability already at 25 μM (85.03% ± 1.99), decreasing to 74.78% ± 1.58 at 50 μM, 46.69% ± 3.08 at 75 μM, and 26.41% ± 2.17 at 100 μM. Using BzTz at a concentration of 100 μM as a reference for the parent compound, **MG-1** exceeded the maximum effect of BzTz starting at 50 μM, indicating a more potent activity profile following derivatization. **MG-2** showed a slight reduction in cell viability starting at 50 μM (87.15% ± 1.8), reaching 77.66% ± 1.58 at 75 μM and 75.44% ± 0.56 at 100 μM. Compared to the reference effect of BzTz at 100 μM, **MG-2** showed only a limited improvement, observed mainly at higher concentrations. **MG-3** showed a concentration-dependent decrease in cell viability, evidenced by a decrease in cell viability from 76.58% ± 1.31 at 25 μM to 34.81% ± 4.2 at 100 μM. Compared to the maximum effect observed for BzTz, **MG-3** also exhibited a significantly improved activity profile in A375 cells. **MG-4** had a slight impact on cell viability at 25 μM (90.03% ± 3.38), becoming 87.94% ± 2.48 at 50 μM, 86.9% ± 5.67 at 75 μM, and 78.11% ± 8.34 at 100 μM. **MG-4** did not demonstrate a significantly improved effect compared to the maximum response observed with BzTz. Overall, **MG-1** and **MG-3** were the most active compounds in A375 cells (Figure 3).

**Figure 4:**
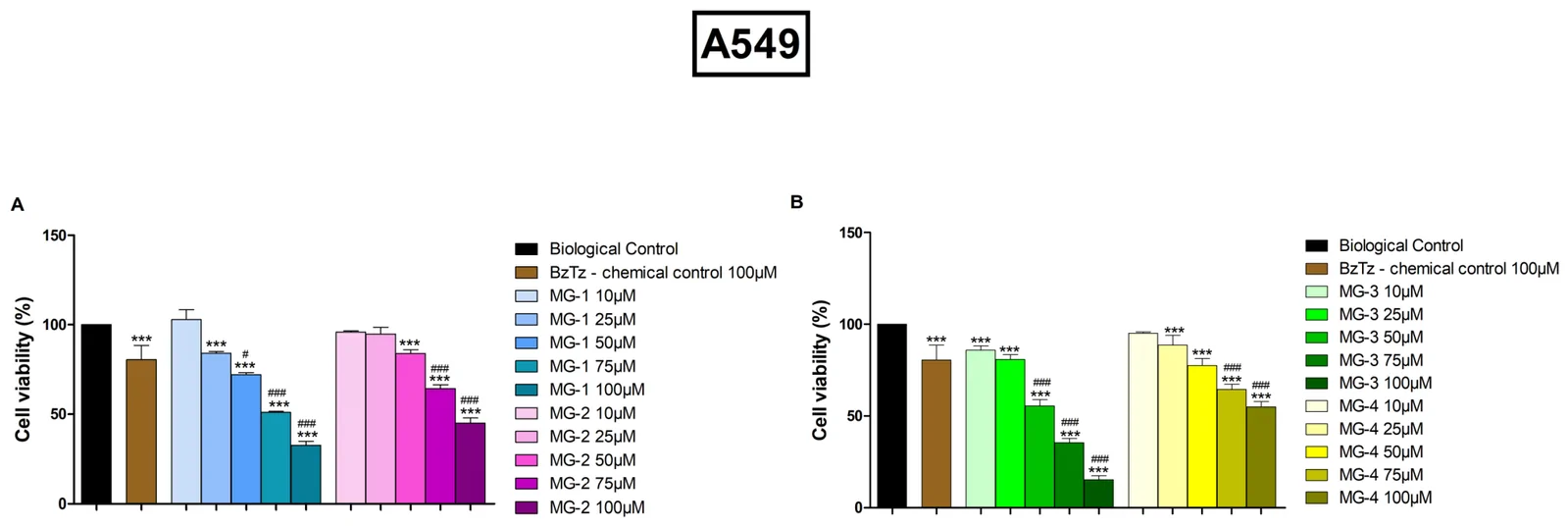
Viability of A549 cells after 48 h of treatment with compounds **MG-1**, **MG-2**, **MG-3**, and **MG-4** at concentrations of 10, 25, 50, 75, and 100 μM. (**A**) Cell viability following treatment with **MG-1** and **MG-2**; (**B**) Cell viability following treatment with **MG-3** and **MG-4**. BzTz at 100 μM was included as the reference condition for the parent scaffold. Cell viability was assessed using the Alamar Blue assay and expressed as percentage relative to the biological control (100%). Data represent mean ± SD of three independent experiments performed in triplicate. Differences were considered statistically significant at *p* < 0.05 (*** *p* < 0.001) compared to the biological control. Hash symbols indicate statistically significant differences relative to benzotriazole tested at 100 μM, which was used as the reference condition for the parent scaffold because no IC_50_ value was reached within the tested concentration range (# *p* < 0.05, and ### *p* < 0.001).

With regard to the A549 cell line, the parent compound BzTz exhibited cell viability of 80.47% ± 8.02 compared to the biological control (100%) at the tested concentration of 100 μM. For the **MG-1** compound, cell viability decreased progressively with increasing concentration. At 25 μM, viability was 84.06% ± 0.92, decreasing to 72.04% ± 1.07 at 50 μM, 50.98% ± 0.58 at 75 μM, and 32.67% ± 2.26 at 100 μM, relative to the control (100%). Using the effect of BzTz at a concentration of 100 μM as a reference for the initial structure, **MG-1** produced a stronger viability-reducing effect starting at 50 μM. The **MG-2** compound showed a concentration-dependent reduction in viability starting at 50 μM, where cell viability was 83.99% ± 2.09, decreasing to 64.41% ± 1.99 at 75 μM, and reaching 44.89% ± 3.11 at 100 μM compared to the control (100%). Compared to the maximum effect observed for BzTz, **MG-2** also exhibited enhanced activity at higher concentrations. The **MG-3** compound was the most active derivative in A549 cells, showing effects even at 10 μM (85.78% ± 2.28), reaching 80.69% ± 2.7 at 25 μM, 55.42% ± 3.41 at 50 μM, 35.15% ± 2.49 at 75 μM, and 15.11% ± 2.27 at 100 μM compared to the control (100%). In particular, **MG-3** exceeded the maximum effect of BzTz over a wide range of concentrations, confirming a significantly improved activity profile following derivatization. The **MG-4** compound showed an intermediate viability-reducing effect starting at 25 μM (88.44% ± 5.48), decreasing to 77.45% ± 3.85 at 50 μM, 64.23% ± 3 at 75 μM, and 54.82% ± 2.92 at 100 μM. **MG-4** also exceeded the maximum effect observed for BzTz at higher concentrations, although its activity remained lower than that of **MG-1** and **MG-3**. Overall, these results indicate that A549 cells were particularly sensitive to **MG-3**, followed by **MG-1** (Figure 4).

**Figure 5:**
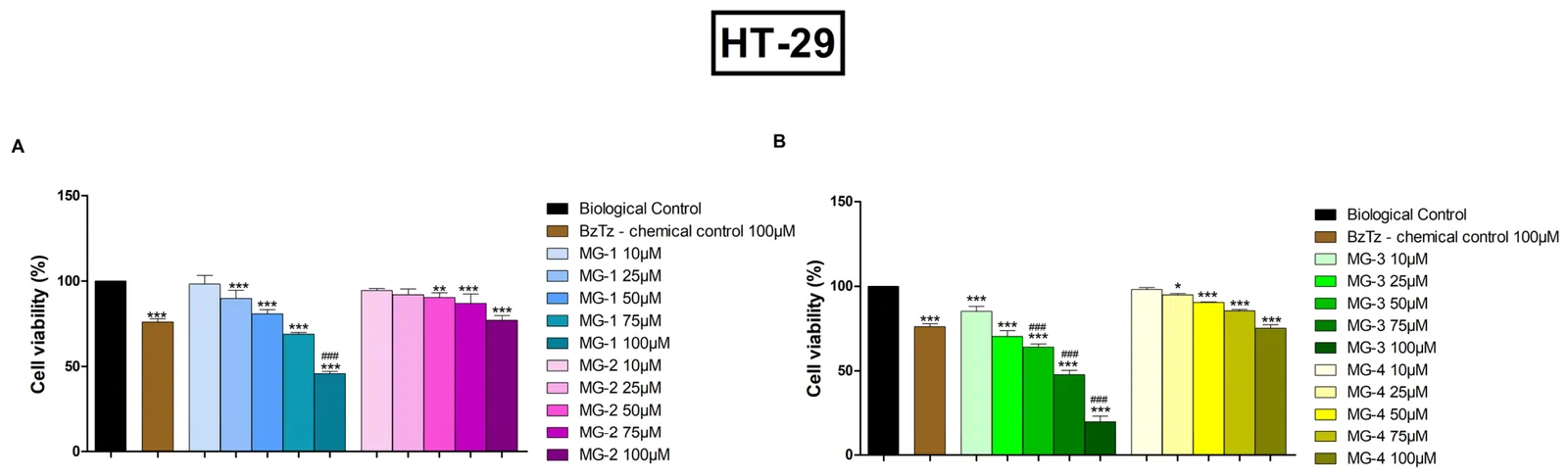
Viability of HT-29 cells after 48 h of treatment with compounds **MG-1**, **MG-2**, **MG-3**, and **MG-4** at concentrations of 10, 25, 50, 75, and 100 μM. (**A**) Cell viability following treatment with **MG-1** and **MG-2**; (**B**) Cell viability following treatment with **MG-3** and **MG-4**. BzTz at 100 μM was included as the reference condition for the parent scaffold. Cell viability was assessed using the Alamar Blue assay and expressed as percentage relative to the biological control (100%). Data represent mean ± SD of three independent experiments performed in triplicate. Differences were considered statistically significant at *p* < 0.05 (* *p* < 0.05, ** *p* < 0.01 and *** *p* < 0.001) compared to the biological control. Hash symbols indicate statistically significant differences relative to benzotriazole tested at 100 μM, which was used as the reference condition for the parent scaffold because no IC_50_ value was reached within the tested concentration range (### *p* < 0.001).

In the HT-29 colorectal adenocarcinoma cell line, the parent compound BzTz exhibited cell viability of 76.08% ± 1.85 compared to the biological control (100%) at the tested concentration of 100 μM. The **MG-1** compound exhibited a concentration-dependent reduction in cell viability, reducing cell viability to 89.78% ± 4.82 at 25 μM, 80.83% ± 2.37 at 50 μM, 68.92% ± 0.95 at 75 μM, and 45.74% ± 1.35 at 100 μM, compared to the control (100%). Compared to the maximum effect of BzTz at a concentration of 100 μM, **MG-1** exhibited stronger activity at concentrations of 75 and 100 μM. The **MG-2** compound showed only a slight effect on viability starting at 50 μM (90.33% ± 2.83), decreasing to 86.97% ± 5.51 at 75 μM, and 77.03% ± 2.78 at 100 μM compared to the control (100%). Compared to the maximum response of BzTz, **MG-2** did not demonstrate a significant improvement in activity. The **MG-3** compound showed a marked concentration-dependent reduction in cell viability already at 10 μM (85.26% ± 2.9), reaching 70.43% ± 3.25 at 25 μM, 64.12% ± 1.88 at 50 μM, 47.72% ± 2.52 at 75 μM, and 19.96% ± 3.32 at 100 μM compared to the control (100%). **MG-3** far exceeded the maximum effect of BzTz and proved to be the most active derivative in HT-29 cells. The **MG-4** compound showed a dose-dependent but comparatively weak effect, with cell viability of 94.92% ± 0.86 at 25 μM, 90.47% ± 0.4 at 50 μM, 85.48% ± 0.97 at 75 μM, and 75.21% ± 2.01 at 100 μM compared to the control (100%). Similarly, **MG-4** did not show a significantly improved activity profile compared to the maximum response observed for BzTz. Overall, **MG-3** remained the most active derivative in HT-29 cells, while **MG-1** exhibited moderate activity. **MG-2** and **MG-4** showed only a limited improvement over the original compound (Figure 5).

### 2.4. The Effects of Benzotriazole and Its Hybrids on Cell Morphology

**Figure 6:**
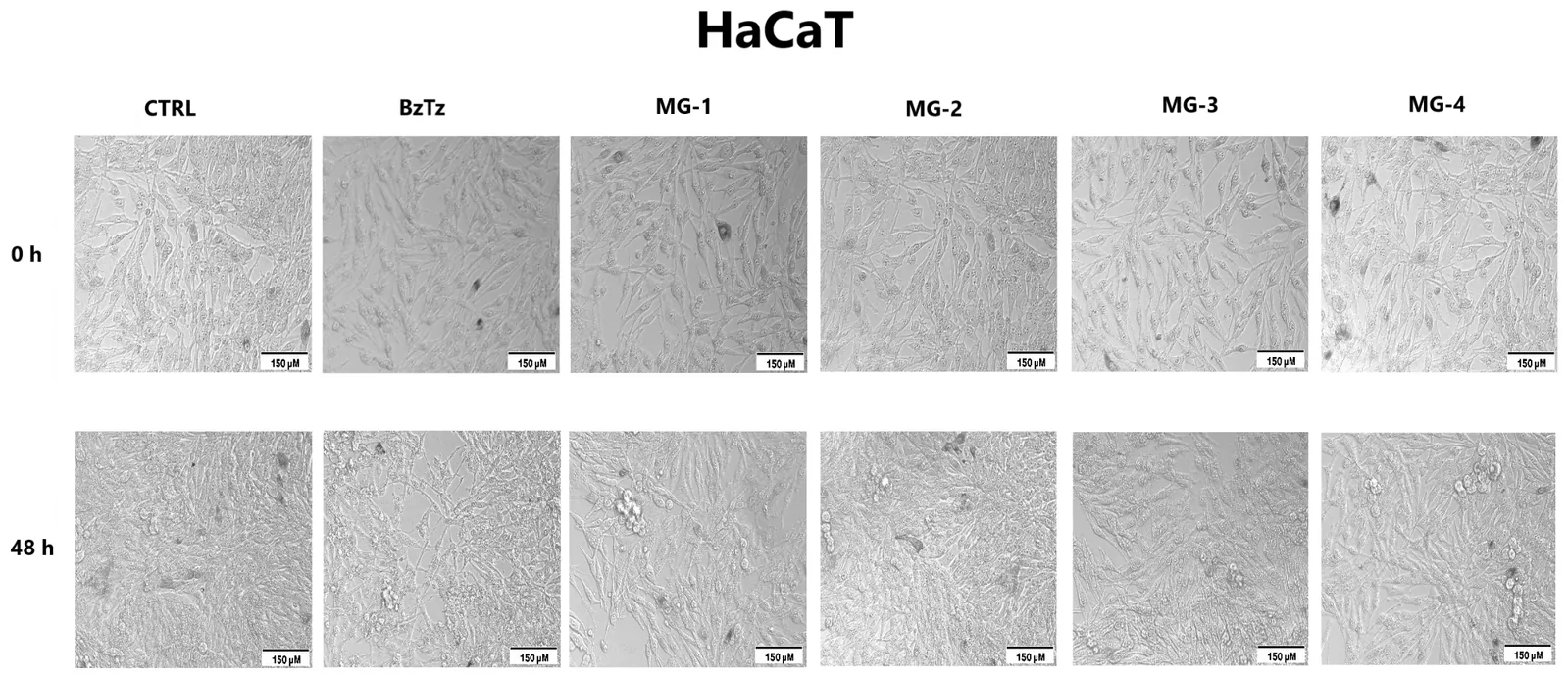
Effects of BzTz, **MG-1**, **MG-2**, **MG-3** and **MG-4** on the morphology of human keratinocyte HaCaT cells, both at 0 h and 48 h after stimulation; the measurement scale was 150 μm.

**Figure 7:**
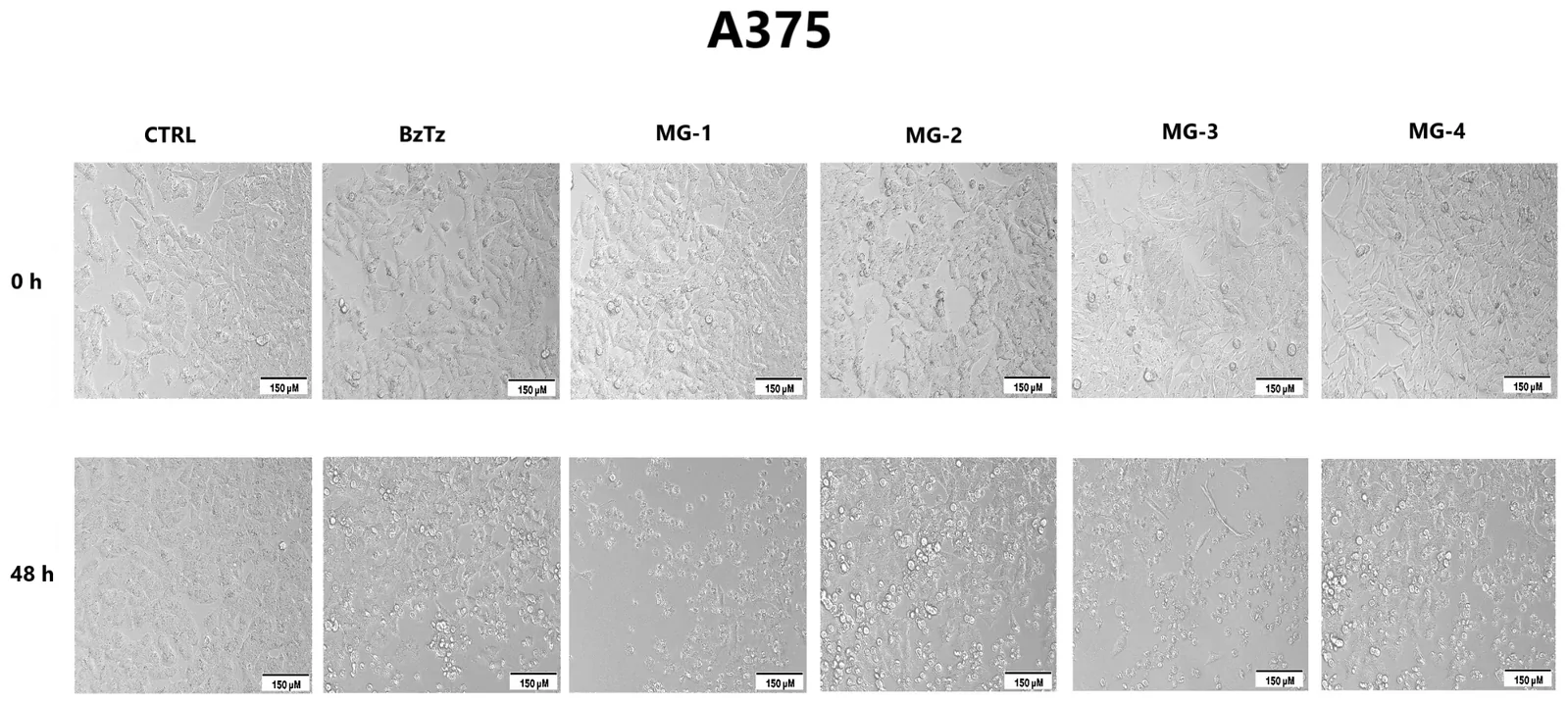
Effects of BzTz, **MG-1**, **MG-2**, **MG-3** and **MG-4** on the morphology of human melanoma A375 cells, both at 0 h and 48 h after stimulation; the measurement scale was 150 μm.

**Figure 8:**
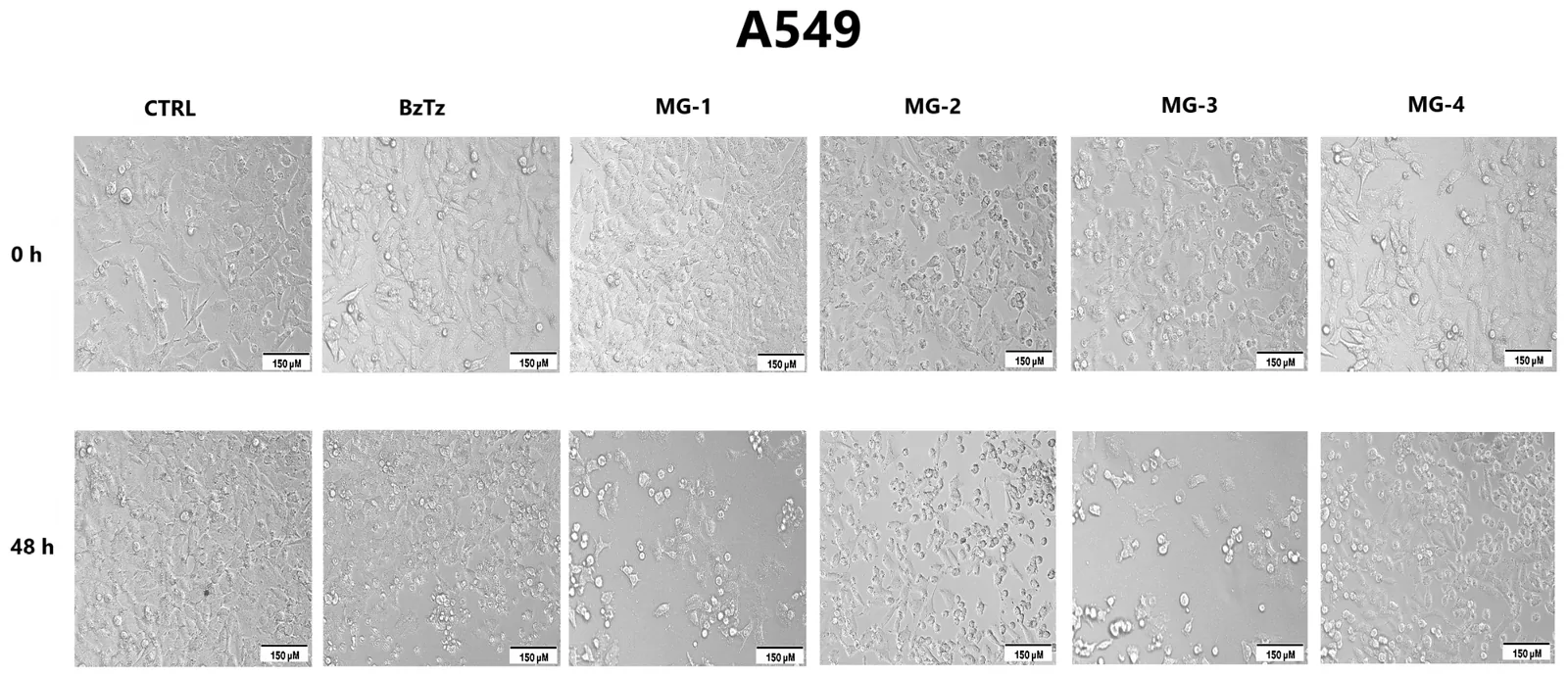
Effects of BzTz, **MG-1**, **MG-2**, **MG-3** and **MG-4** on the morphology of human non-small cell lung cancer A549 cells, both at 0 h and 48 h after stimulation; the measurement scale was 150 μm.

**Figure 9:**
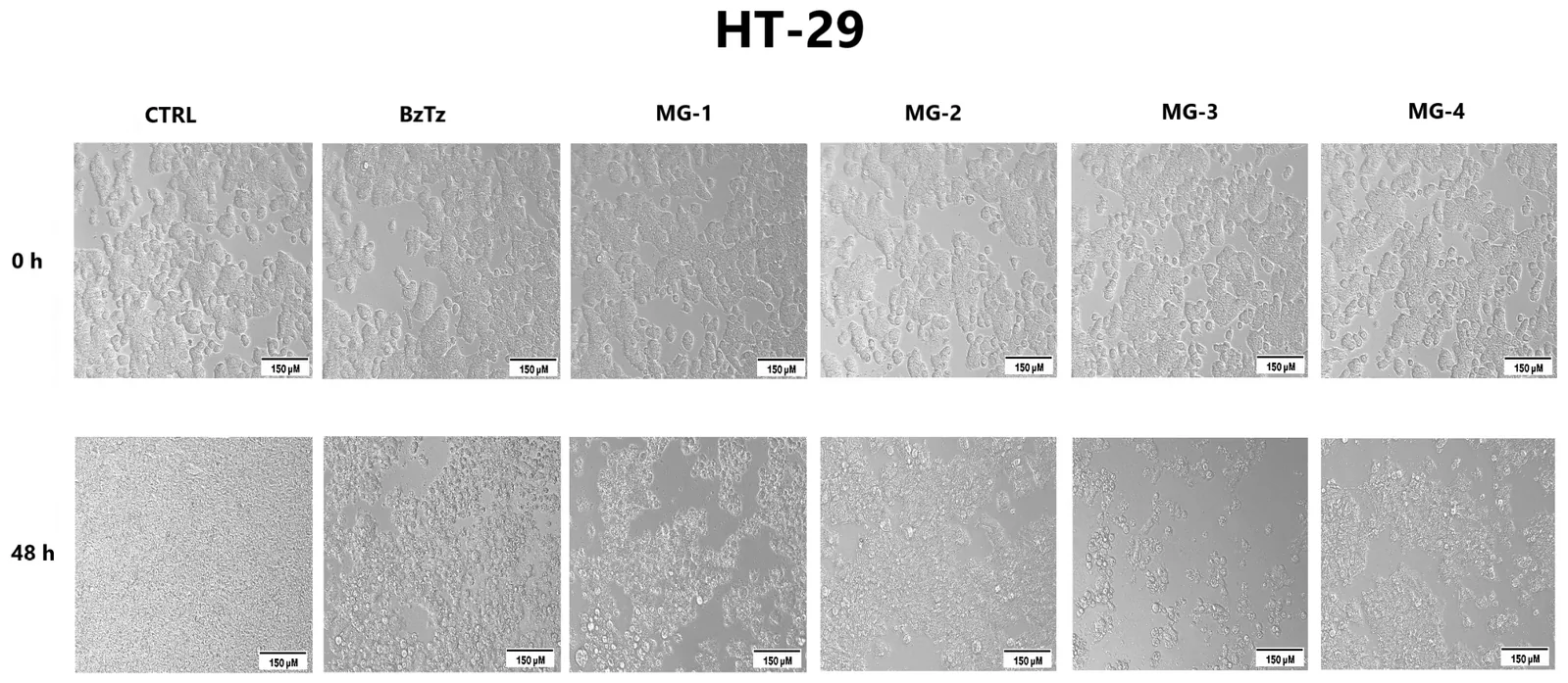
Effects of BzTz, **MG-1**, **MG-2**, **MG-3** and **MG-4** on the morphology of colorectal adenocarcinoma HT-29 cells, both at 0 h and 48 h after stimulation; the measurement scale was 150 μm.

Morphological changes were assessed in all four cell lines (HaCaT, A375, A549, and HT-29) following treatment with the four compounds **MG-1**, **MG-2**, **MG-3**, and **MG-4**, in accordance with the cell viability results. An assessment of morphological changes for the intermediate derivatives (**1a**–**d**) was not performed because the initial screening did not reveal biologically significant reductions in cell viability. Treatment was applied either at the IC_50_ value determined in the viability assays or at the highest test concentration of 100 μM. Changes such as cell rounding, detachment from the substrate, and cell density were monitored throughout this test. All of these changes were evaluated relative to the control group. These results serve to complete the viability data and the corresponding morphological observations are presented in Figure 6, Figure 7, Figure 8 and Figure 9.

### 2.5. Immunofluorescence-Based Evaluation of Treatment-Induced Nuclear and Cytoskeletal Changes

**Figure 10:**
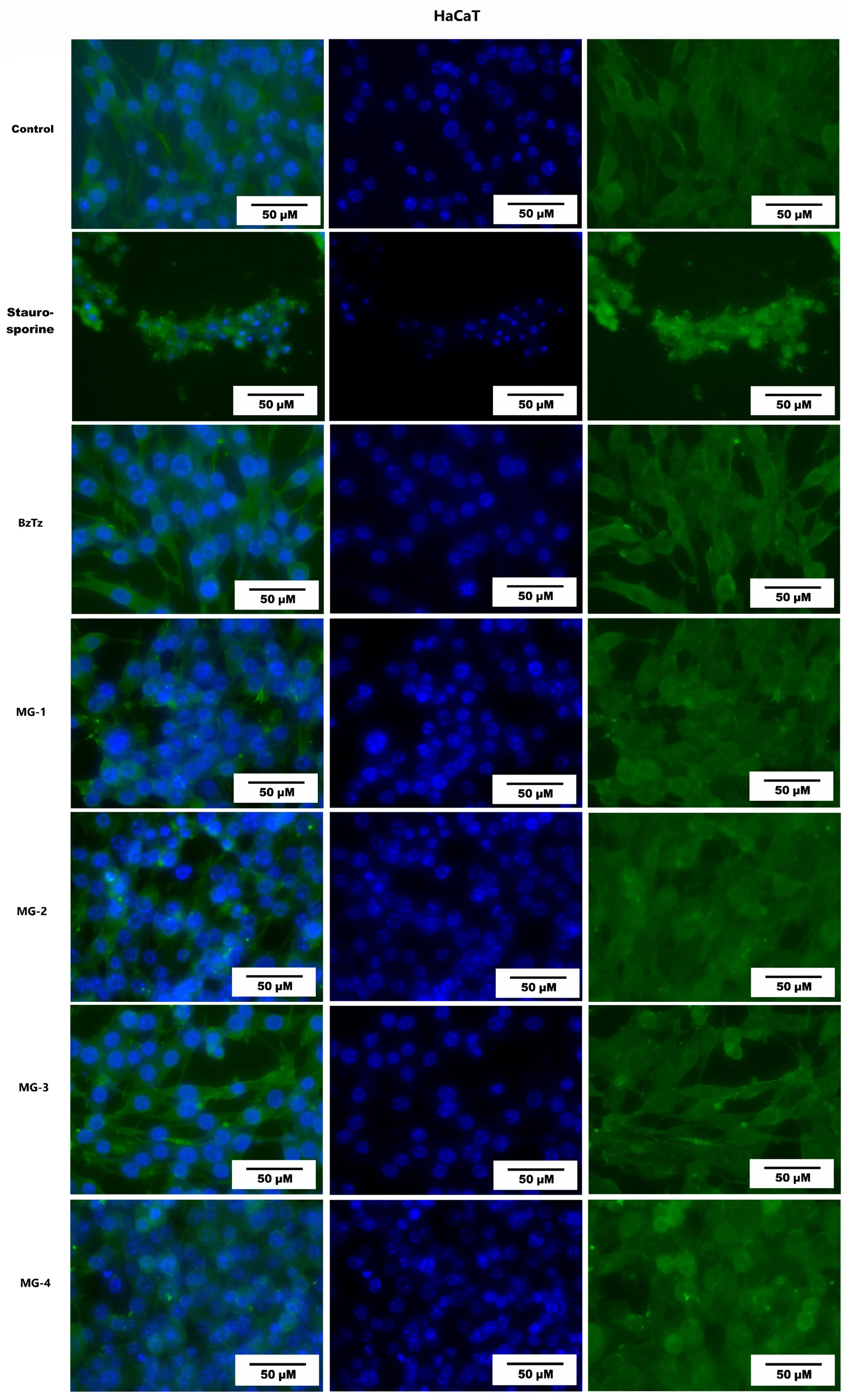
HaCaT results following both 48-h treatment with BzTz, **MG-1**, **MG-2**, **MG-3**, and **MG-4** and immunofluorescence staining. Cell nuclei are highlighted in blue, and β-actin in green. Scale bar = 50 μm. Staurosporine (5 μM, positive control).

**Figure 11:**
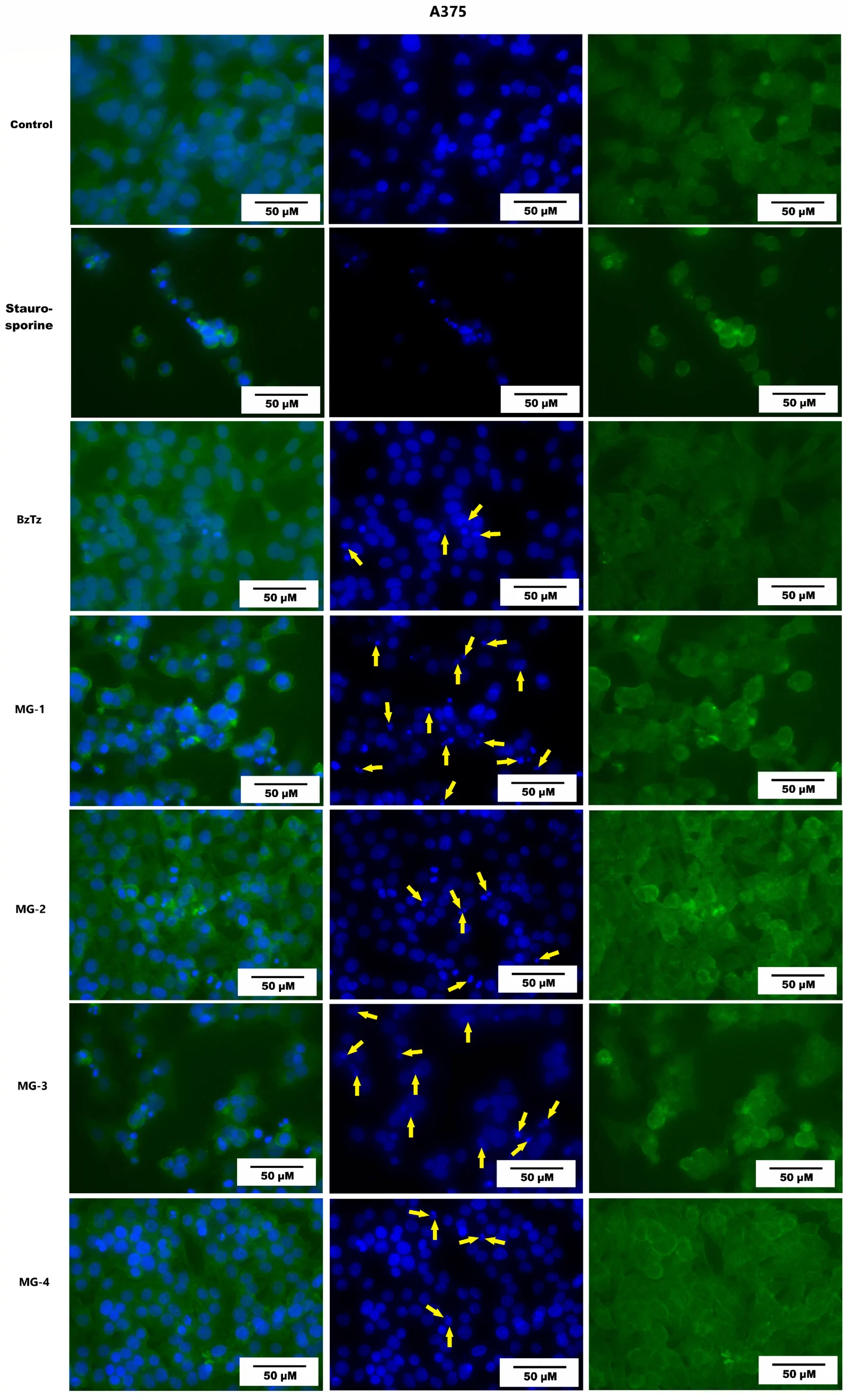
A375 results following both 48-h treatment with BzTz, **MG-1**, **MG-2**, **MG-3**, and **MG-4** and immunofluorescence staining. Cell nuclei are highlighted in blue, and β-actin in green. Scale bar = 50 μm. Staurosporine (5 μM, positive control). Yellow arrows indicate representative nuclear alterations.

**Figure 12:**
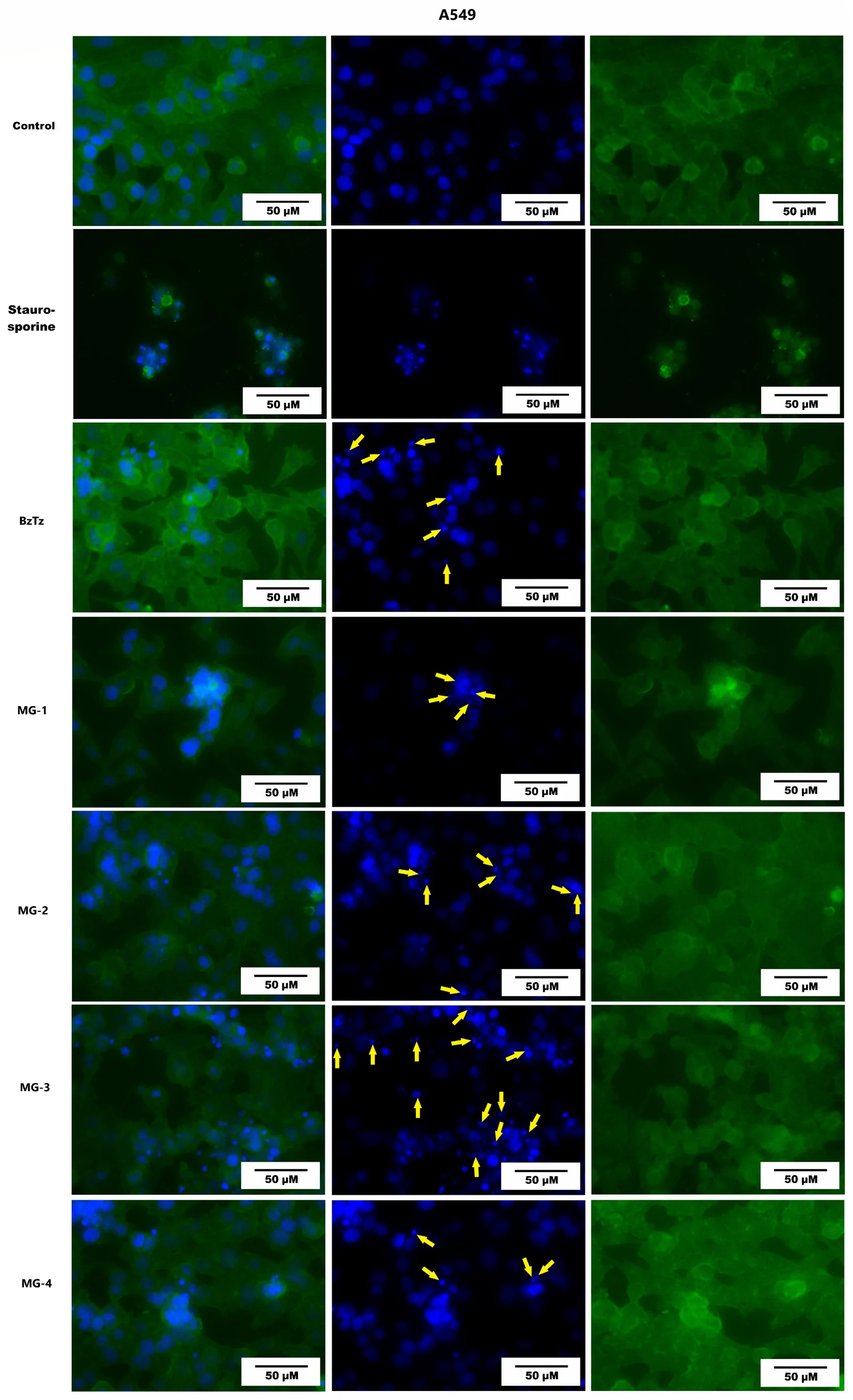
A549 results following both 48-h treatment with BzTz, **MG-1**, **MG-2**, **MG-3**, and **MG-4** and immunofluorescence staining. Cell nuclei are highlighted in blue, and β-actin in green. Scale bar = 50 μm. Staurosporine (5 μM, positive control). Yellow arrows indicate representative nuclear alterations.

**Figure 13:**
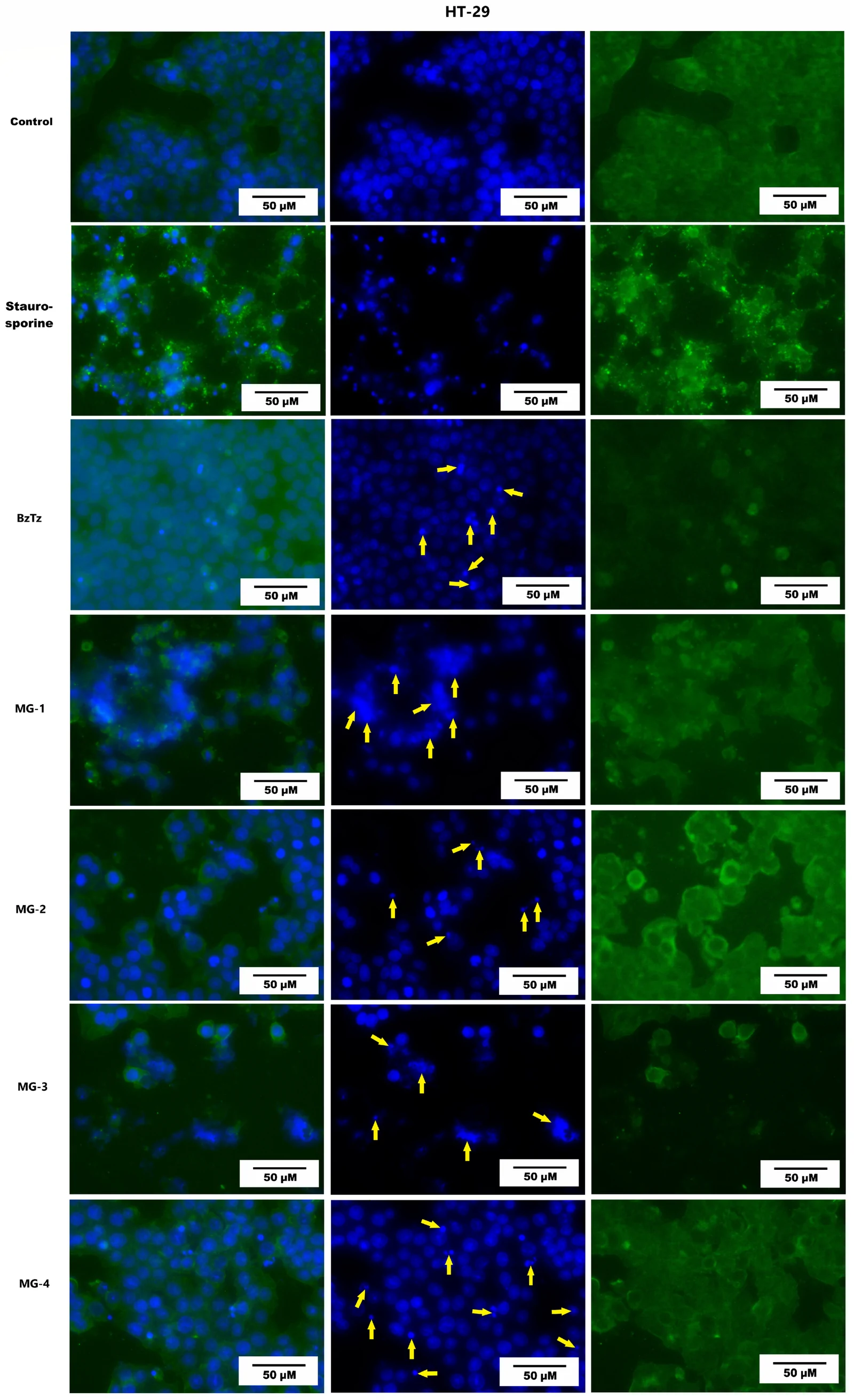
HT-29 results following both 48-h treatment with BzTz, **MG-1**, **MG-2**, **MG-3**, and **MG-4** and immunofluorescence staining. Cell nuclei are highlighted in blue, and β-actin in green. Scale bar = 50 μm. Staurosporine (5 μM, positive control). Yellow arrows indicate representative nuclear alterations.

To study cellular morphological changes, a protocol was applied that involved staining the nuclei with Hoechst and labeling the cytoskeleton using a combination of β-actin and Alexa Fluor 488. This protocol was applied to all four cell lines included in the study (HaCaT, A375, A549, and HT-29), which had previously been treated for 48 h with the IC_50_ derived from cell viability data or with the maximum tested concentration of 100 μM when IC_50_ values could not be determined, of the compounds BzTz, **MG-1**, **MG-2**, **MG-3**, and **MG-4**. This protocol was not applied to the synthetic intermediates (**1a**–**d**), as they did not demonstrate significant biological cytotoxicity in the initial screening. According to the data presented in Figure 10, human keratinocytes largely retained their initial morphological characteristics (intact nuclei and well-organized actin filaments, similar to those observed in the control) following the 48-h treatment, when treated with BzTz and the **MG-1**–**MG-4** series, explicitly excluding the positive control (staurosporine). In contrast, the results for the other three cancer cell lines (A375, A549, and HT-29) showed morphological changes comparable to cellular apoptosis after 48 h of treatment (Figure 11, Figure 12 and Figure 13). In all three cancer cell lines, the most striking morphological changes occurred with treatment using **MG-1** and **MG-3**, where cellular morphology revealed nuclear shrinkage, chromatin fragmentation, the formation of membrane vesicles, and disruption of the cytoskeletal network. Morphological changes such as reduced adhesion, disrupted actin organization, and condensed nuclei can also be observed following treatment with **MG-2** and **MG-4**.

### 2.6. Molecular Docking

Molecular docking was performed for compounds **MG-1**–**MG-4** against a panel of eight cancer-related protein targets, namely EGFR, BRAF^V600E, MEK1, PI3Kα, AKT1, FAK, BCL-2, and BCL-xL. The corresponding co-crystallized ligands were included as reference compounds. The docking scores obtained for the investigated compounds and native ligands are presented in Table 2.

Among the investigated targets, MEK1 (PDB ID: 3DV3) showed the most consistent favourable docking profile for the **MG** series. The predicted binding energies ranged from −9.0 to −9.4 kcal/mol, compared with −9.4 kcal/mol for the co-crystallized ligand PF-04622664. **MG-1** displayed a docking score of −9.4 kcal/mol, whereas **MG-3** and **MG-4** showed values of −9.2 kcal/mol and **MG-2** a value of −9.0 kcal/mol. Favourable docking scores were also obtained for several compounds against EGFR and AKT1, while larger differences relative to the corresponding native ligands were observed for BRAF^V600E, FAK, BCL-2, and BCL-xL.

**Figure 14:**
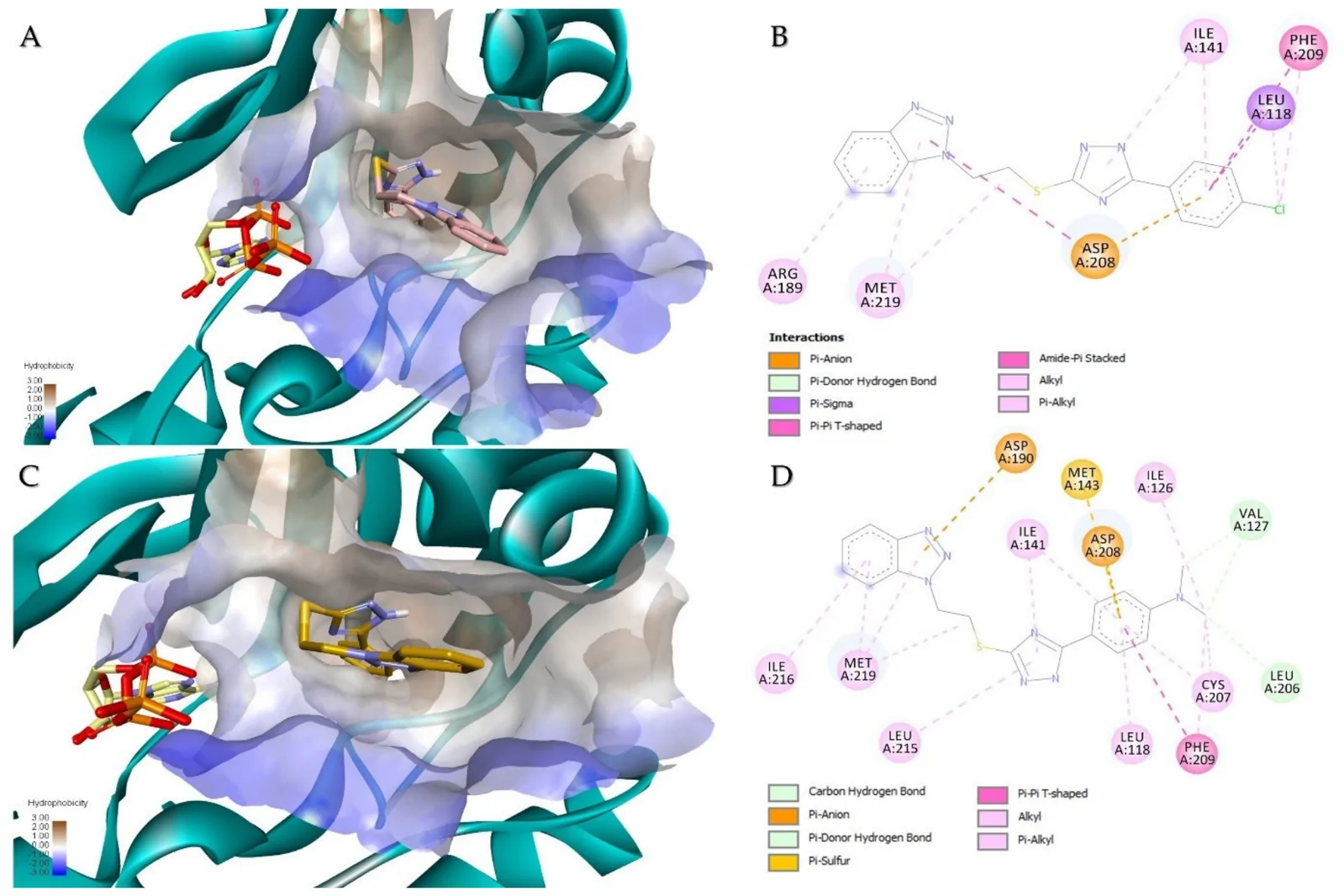
Predicted binding modes of **MG-1** and **MG-3** in the MEK1 binding pocket (PDB ID: 3DV3). (**A**) Three-dimensional binding pose of **MG-1**. (**B**) Two-dimensional interaction diagram of **MG-1**. (**C**) Three-dimensional binding pose of **MG-3**. (**D**) Two-dimensional interaction diagram of **MG-3**.

Based on the docking results and the experimental cell-viability data, **MG-1** and **MG-3** were selected for further analysis in complex with MEK1. Both compounds occupied the binding pocket defined by the co-crystallized ligand and established multiple contacts with residues located within the MEK1 binding site (Figure 14). **MG-1** interacted mainly with Ile141, Leu118, Phe209, Met219, Arg189, and Asp208, the latter being involved in a π-anion interaction. **MG-3** displayed a broader interaction pattern involving Ile141, Ile126, Val127, Leu118, Leu206, Leu215, Ile216, Met219, Cys207, Phe209, Asp190, Asp208, and Met143, comprising hydrophobic/π contacts together with π-anion and π-sulfur interactions.

The MEK1–**MG-1** and MEK1–**MG-3** complexes were subsequently subjected to 100 ns molecular dynamics simulations, followed by MM/GBSA analysis, to further evaluate the stability of the predicted protein–ligand complexes.

### 2.7. Molecular Dynamics Simulations and MM-GBSA Analysis

**Figure 15:**
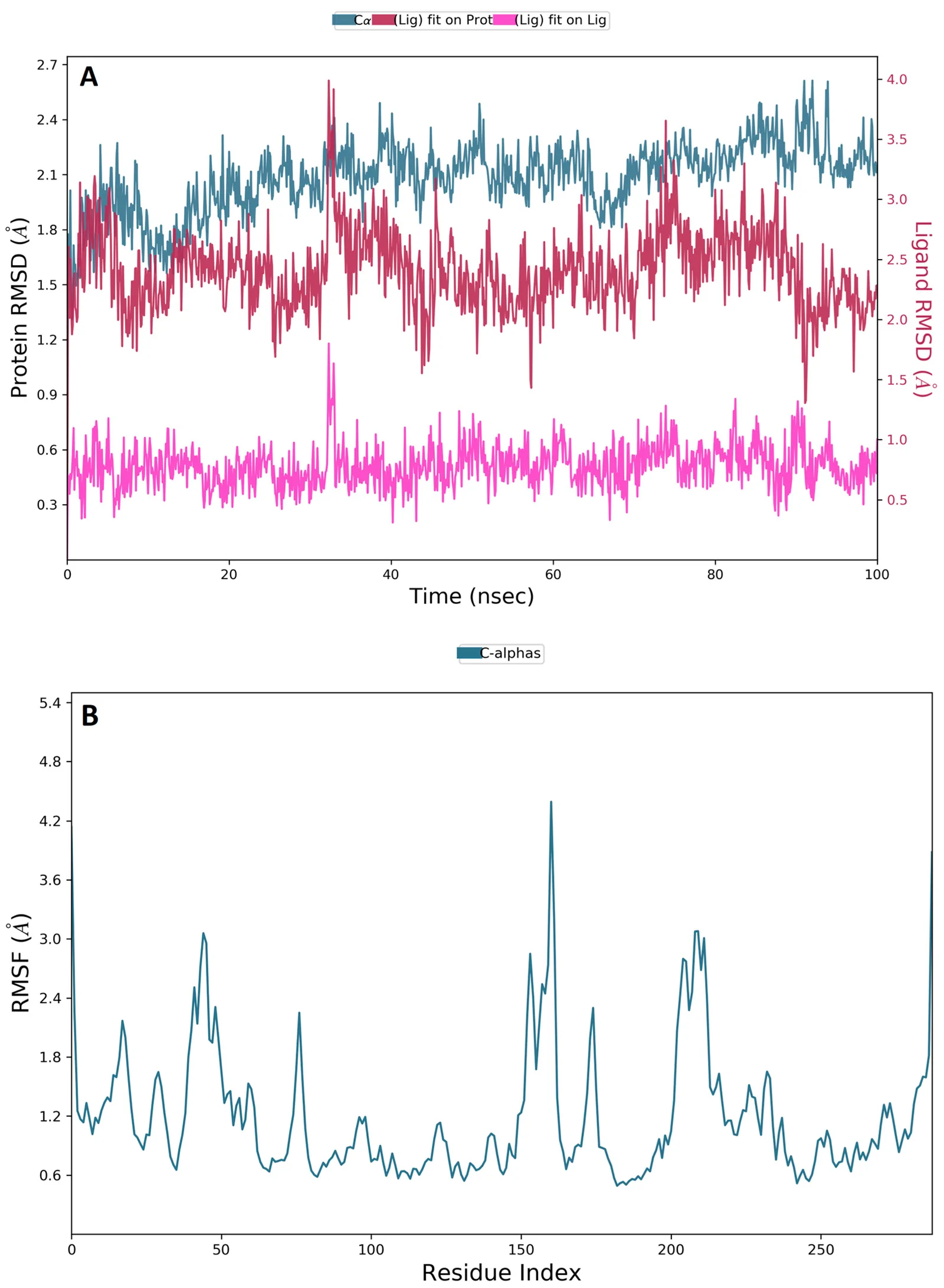

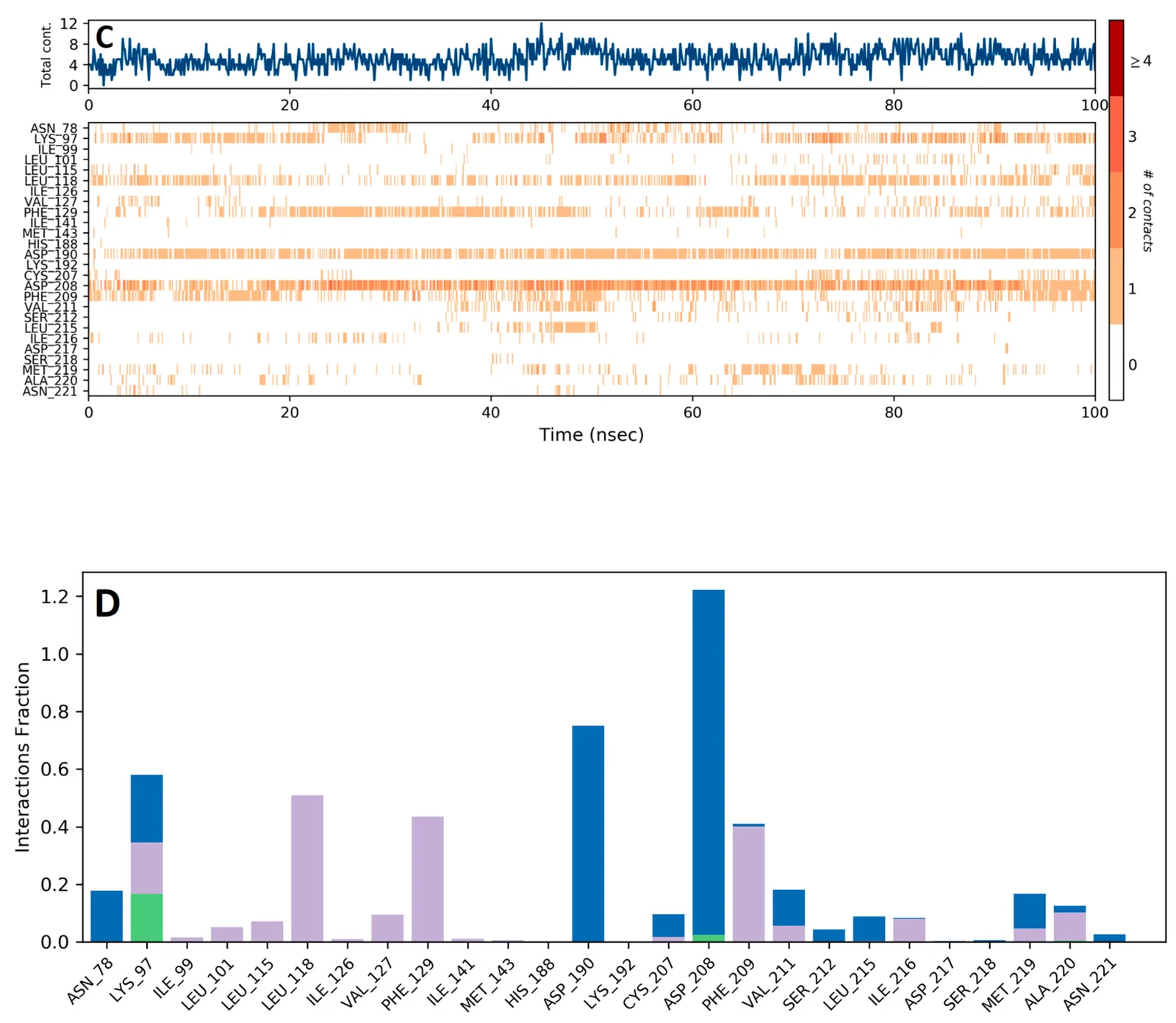
Molecular dynamics analysis of the MEK1–**MG-1** complex over a 100 ns simulation. (**A**) Root-mean-square deviation (RMSD) profiles of the protein Cα atoms and **MG-1**, including ligand RMSD after fitting on the protein and on the ligand. (**B**) Root-mean-square fluctuation (RMSF) profile of MEK1 Cα atoms. (**C**) Timeline of protein–ligand contacts and total number of contacts formed by **MG-1** throughout the simulation. (**D**) Interaction fractions of MEK1 residues involved in contacts with **MG-1** during the 100 ns trajectory.

To further evaluate the stability of the predicted docking pose, the MEK1–**MG-1** complex was subjected to a 100 ns molecular dynamics simulation. The protein Cα RMSD remained within approximately 1.8–2.4 Å throughout the simulation, indicating overall structural stability of the complex. The ligand RMSD calculated after fitting on the protein showed moderate fluctuations, whereas the ligand RMSD calculated after fitting on the ligand remained low, indicating that **MG-1** preserved a stable internal conformation during the simulation (Figure 15A). The protein Cα RMSF profile showed limited fluctuations for most residues, with higher mobility observed mainly in loop regions and terminal segments (Figure 15B). Analysis of the protein–ligand contacts revealed persistent interactions involving several binding-site residues, among which Asp208, Asp190, Phe209, Leu118, Lys97, and Phe129 were the most prominent. In particular, Asp208 displayed the highest interaction frequency during the simulation. These results supported the stability of the MEK1–**MG-1** complex throughout the 100 ns simulation period (Figure 15C,D).

**Figure 16:**
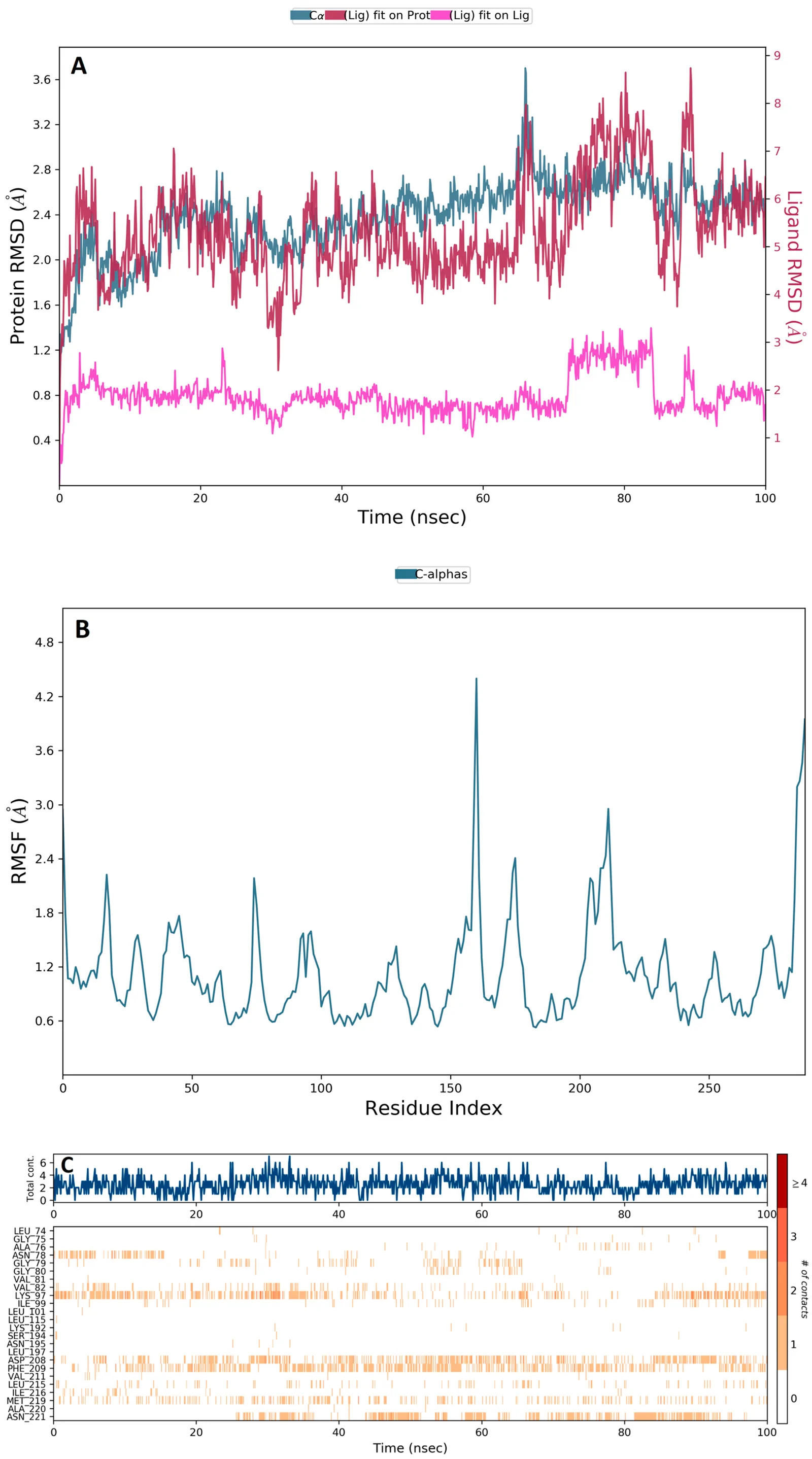

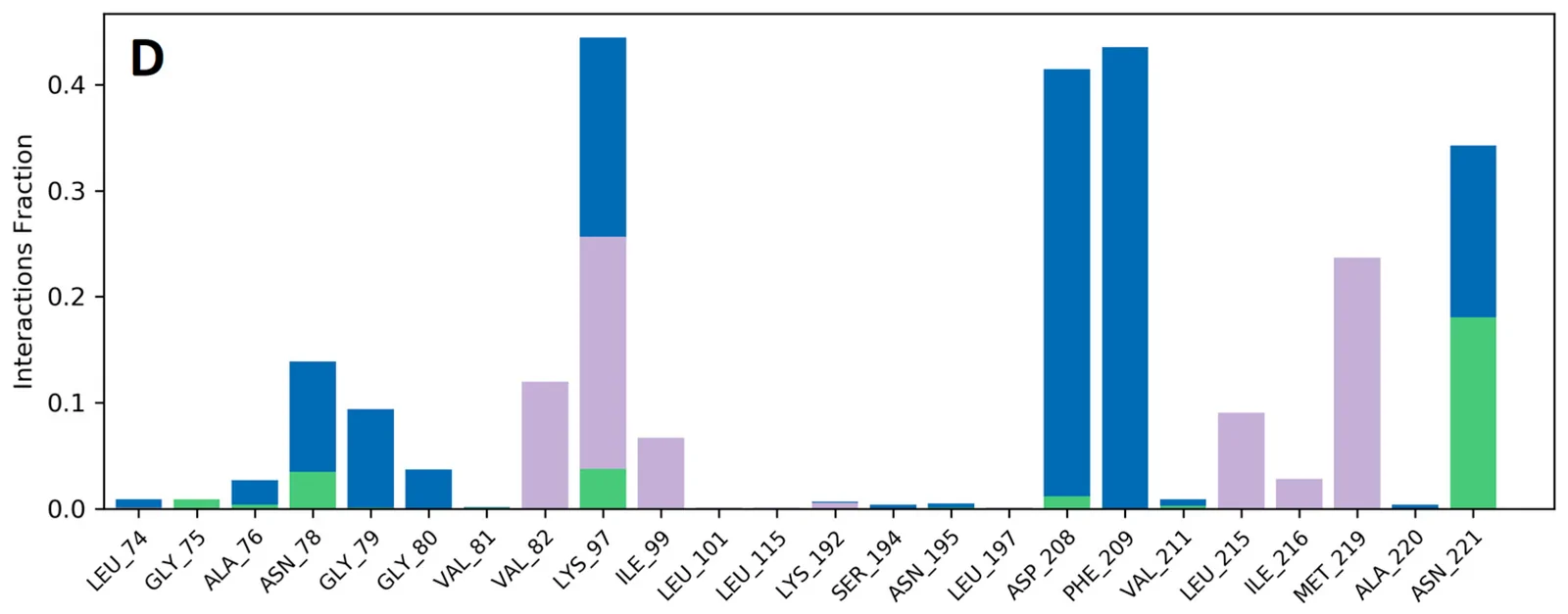
Molecular dynamics analysis of the MEK1–**MG-3** complex over a 100 ns simulation. (**A**) Root-mean-square deviation (RMSD) profiles of the protein Cα atoms and **MG-3**, including ligand RMSD after fitting on the protein and on the ligand. (**B**) Root-mean-square fluctuation (RMSF) profile of MEK1 Cα atoms. (**C**) Timeline of protein–ligand contacts and total number of contacts formed by **MG-3** throughout the simulation. (**D**) Interaction fractions of MEK1 residues involved in contacts with **MG-3** during the 100 ns trajectory.

The MEK1–**MG-3** complex was also subjected to a 100 ns molecular dynamics simulation. The protein Cα RMSD generally remained within approximately 1.6–2.8 Å, with transient increases observed during the second half of the simulation. The ligand RMSD calculated after fitting on the protein showed more pronounced fluctuations, remaining mostly within approximately 4–6 Å and temporarily increasing to around 7–8 Å between approximately 70 and 85 ns. The ligand RMSD calculated after fitting on the ligand remained considerably lower, generally around 1.5–2.0 Å, with a transient increase to approximately 3 Å over the same interval, indicating positional and conformational rearrangements of **MG-3** during the simulation (Figure 16A).

The protein Cα RMSF profile showed relatively limited fluctuations for most residues, while higher mobility was observed in several flexible regions and near the terminal segments (Figure 16B). Protein–ligand contact analysis showed that interactions were maintained throughout the 100 ns trajectory. The highest interaction fractions were observed for Lys97, Asp208, Phe209, Asn221, and Met219, with additional recurrent contacts involving residues such as Asn78, Gly79, Val82, Ile99, Leu215, and Ile216. The persistent contact pattern indicates that **MG-3** remained associated with the MEK1 binding region despite the positional rearrangements observed in the ligand RMSD profile (Figure 16C,D).

MM-GBSA analysis revealed a more favorable predicted binding free energy for the MEK1–**MG-1** complex (ΔG_bind = −54.83 kcal/mol) compared with MEK1–**MG-3** (ΔG_bind = −40.70 kcal/mol) (Table 3). For **MG-1**, the main favorable energetic contributions were the van der Waals (−45.53 kcal/mol), lipophilic (−22.96 kcal/mol), and Coulombic (−16.66 kcal/mol) terms, whereas the generalized Born solvation contribution was unfavorable (+26.73 kcal/mol).

**MG-3** showed more favorable van der Waals (−50.90 kcal/mol), Coulombic (−23.30 kcal/mol), and hydrogen-bonding (−1.66 kcal/mol) contributions than **MG-1**. However, this complex displayed a higher unfavorable solvation contribution (+39.45 kcal/mol) and a less favorable lipophilic contribution (−10.89 kcal/mol), resulting in a less negative overall ΔG_bind. Ligand efficiency was −2.28 kcal/mol for **MG-1** and −1.57 kcal/mol for **MG-3**.

Overall, both **MG-1** and **MG-3** remained associated with the MEK1 binding region throughout the 100 ns simulations. However, **MG-1** showed lower ligand positional fluctuations and a more favourable MM-GBSA binding energy, whereas **MG-3** underwent more pronounced positional rearrangements during the trajectory. Taken together, the MD and MM-GBSA results indicate a more stable predicted interaction of **MG-1** with MEK1 under the investigated simulation conditions.

## 3. Discussion

The present study combined the chemical synthesis and structural characterization with the in vitro biological evaluation of a small series of newly synthesized benzotriazole–1,2,4-triazole hybrids. The experimental design was intended to determine whether the derivatization of the benzotriazole scaffold with various aryl–1,2,4-triazole moieties could modify its biological profile, with particular emphasis on its antitumor activity. Among the four newly synthesized compounds, **MG-1** and **MG-3** emerged as the most active compounds, although their potency remained moderate and their effects varied across the A375, A549, and HT-29 cells.

From a chemical perspective, the synthetic strategy successfully provided four new benzotriazole–1,2,4-triazole hybrids in which the two heterocyclic systems are connected through a flexible 2-sulfanylethyl linker. The final compounds were obtained in variable, generally moderate yields, ranging from 21% for **MG-4** to 51.4% for **MG-3**, while **MG-1** and **MG-2** were isolated in comparable yields of approximately 36%. Although these differences indicate that the nature of the terminal aryl substituent may influence the overall synthetic outcome, the present series is too limited to establish a direct relationship between the substituents’ electronic properties and reaction yield. Moreover, the reaction yield may reflect several factors, including reaction conversion, competing processes, solubility, and chromatographic recovery. Therefore, the synthetic results primarily demonstrate the feasibility of the adopted strategy for generating structurally related benzotriazole–1,2,4-triazole hybrids rather than a defined substituent-dependent reactivity pattern.

An additional chemically relevant feature was observed during NMR characterization. In DMSO-d_6_, several signals of **MG-1**–**MG-4** appeared broadened or duplicated, whereas the addition of trifluoroacetic acid resulted in a single set of sharper resonances. Similar NMR behavior has previously been described for 3,5-disubstituted 1,2,4-triazoles and has been attributed to slowly exchanging tautomeric forms [23,24]. The collapse of the broadened or duplicated signals after acidification is therefore consistent with the acceleration of the tautomeric exchange on the NMR timescale. Comparable behavior has also been reported for related 5-substituted 1,2,4-triazole derivatives [22]. Together with the characteristic ^1^H and ^13^C NMR resonances and FTIR data, these observations support the proposed structures of the newly synthesized hybrids. Importantly, the possible coexistence of tautomeric forms should be regarded primarily as a structural characteristic of the 1,2,4-triazole moiety; the present experiments do not establish whether this equilibrium contributes to the biological effects observed subsequently.

**MG-1**–**MG-4** share the same benzotriazole–ethylsulfanyl–1,2,4-triazole framework and differ mainly by the *para* substituent of the terminal phenyl ring. The differences observed in their biological activity suggest that this substitution may influence the response; however, the limited number of compounds does not allow a formal structure–activity relationship to be established.

From a biological standpoint, we observed a cell line-dependent reduction in cell viability following 48-h treatment with benzotriazole–1,2,4-triazole hybrids. Thus, for the BzTz scaffold, we were unable to obtain an IC_50_ of up to 100 μM for any tumor line, but for the **MG-1** compound, we obtained an IC_50_ of 75.43 ± 2.77 μM in A375 cells, 77.15 ± 1.32 μM in A549 cells, and 96.28 ± 1.72 μM in HT-29 cells. The **MG-3** compound also showed a good response, reaching an IC_50_ of 78.97 ± 3.08 μM in A375, 56.78 ± 2.57 μM in A549, and 69.19 ± 3.97 μM in HT-29, respectively. Only a single IC_50_ value of 90.20 ± 5.09 μM was obtained just for the A549 tumor line for compound **MG-2**, while no IC_50_ value was determined for compound **MG-4** in any tumor line. For this limited series of compounds, **MG-1** proved to be the most active in the A375 cell line, while **MG-3** elicited the best response in A549 and HT-29. These results suggest that derivatization of the scaffold can lead to an enhanced biological response, although this response is strictly dependent on the type of derivative or the tumor cell line and should not be interpreted as a general consequence of hybridization. The Alamar Blue assay involves the quantitative estimation of metabolic viability based on resazurin reduction, where resazurin is an indicator of cellular metabolic activity that cannot distinguish between cytostatic and cytotoxic effects [25]. Accordingly, the present findings should primarily be interpreted as treatment-induced reductions in metabolic viability rather than direct proof of cell death. This distinction is particularly relevant when comparing the final compounds with BzTz under identical experimental conditions. At 100 μM, BzTz yielded viabilities of 85.21% in A375, 80.47% in A549, and 76.08% in HT-29 cells, whereas **MG-1** reduced viability to 26.41%, 32.67%, and 45.74%, and **MG-3** to 34.81%, 15.11%, and 19.96%, respectively. The substantially greater reduction observed with **MG-1** and **MG-3** supports an improved activity profile relative to the parent scaffold under the conditions of this study. However, because only the complete final molecules were tested, the improved response cannot be assigned to a single structural fragment or substituent in isolation. For the keratinocyte cell line, the data obtained from the Alamar Blue assay indicate that we did not achieve an IC_50_ up to 100 μM, with metabolic viability reduced to 78.06% for BzTz, 72.01% for **MG-1**, 76.99% for **MG-2**, 77.55% for **MG-3**, and 77.08% for **MG-4**. However, these results should not be interpreted as an absence of toxicity toward non-tumor cells, but rather merely as lower limits of the selectivity indices, since it is known that IC_50_ values for HaCaT are greater than 100 μM. For **MG-1**, these lower limits are >1.32 for A375, >1.29 for A549, and >1.03 for HT-29, while for **MG-3** they are >1.26, >1.76, and >1.44, respectively. All the values obtained indicate a modest differential sensitivity, with the most pronounced effect observed for **MG-3** in A549. The microscopy data are consistent with the viability findings. In HaCaT cells, nuclei and the actin cytoskeleton were largely preserved after treatment, whereas the tumor cell lines showed reduced adhesion, cytoskeletal disorganization, nuclear condensation, and nuclear fragmentation, with the most pronounced alterations generally observed after **MG-1** and **MG-3** treatment. These findings demonstrate treatment-associated nuclear and cytoskeletal injury that might suggest a pro-apoptotic effect. A structure–activity relationship analysis is limited by the small number of compounds; however, the experimental results on biological activity demonstrate a substituent-dependent response. The *para*-chlorophenyl substituent of compound **MG-1** was the most active against human melanoma and showed comparable activity against the other two tumor lines as the *para*-N,N-dimethylaminophenyl substituent of compound **MG-3**, which proved to be the most active against A549 and HT-29. The *para*-methoxyphenyl derivative found in the **MG-2** compound demonstrated an IC_50_ only on A549; in contrast, for the unsubstituted phenyl derivative of the **MG-4** compound, we did not obtain an IC_50_ below 100 μM. We believe that the terminal aryl substitution could influence the biological results and would be a direction for further chemical exploration if the series is to be expanded, thereby allowing for a better differentiation of the electronic, steric, or physicochemical contributions.

A direct comparative analysis between the antitumor activity recorded in the current study and that reported in the literature is difficult to perform because there are significant differences in both structure and experimental methodology. However, in A375 cells assessed using the Alamar Blue assay at 48 h, Prodea et al. [22] reported IC_50_ values of 22.41 μM and 34.34 μM for Bet-TZ1 and Bet-TZ3, respectively values lower than those obtained for the parent compound (Bet), but also lower than those we obtained for **MG-1** and **MG-3** using the same cell line. Milan et al. [26], using the same cell line and the same viability assay, reported an IC_50_ value of 65.90 μM for betulinic acid and 50.71 μM for the most active liposomal formulation. These comparisons indicate that the compounds in each study exhibited higher activity than the parent compound, similar to the results we obtained under the same experimental conditions, over the same time period, and using the same cell line. Thus, the most active compounds in our study, **MG-1** and **MG-3**, exhibit measurable and moderate antitumor activity. Li et al. reported substantially lower IC_50_ values for other benzotriazole derivatives, including 5.47 μM for their most active compound, but they used a different cell viability assay (CCK) as well as a different structural series [8]. which limits a direct quantitative comparison. Therefore, the most meaningful comparison for the current study remains the internal comparison between the current investigated compounds (**MG-1**–**MG-4**) and BzTz (parent compound) under the same experimental conditions.

To our knowledge, closely related benzotriazole–1,2,4-triazole hybrids have not been previously investigated as MEK1-targeting compounds. Nevertheless, the docking and molecular dynamics results obtained for **MG-1** and **MG-3** showed persistent interactions with residues located within or near the MEK1 allosteric binding region, including Lys97, Asp208, Phe209, and Ile216. Together with the favorable MM-GBSA binding energies, these findings identify MEK1 as a plausible molecular target for this scaffold and provide a rationale for subsequent biochemical validation.

The main limitations of this study are the small number of final compounds, the use of a single non-tumor cell line, the absence of a reference anticancer drug tested in parallel, reliance on a single metabolic viability assay, and the lack of functional experiments capable of defining the mechanism of the nuclear and cytoskeletal changes observed by microscopy. Taken together, the results identify **MG-1** and especially **MG-3** as the most informative compounds for further optimization, but they should be regarded as preliminary lead structures with antitumor potential rather than established antitumor agents. Expansion of the chemical series, determination of selectivity using measured IC_50_ values in several non-tumor cell models, inclusion of reference drugs, and mechanistic assays addressing cell death pathways and target engagement are warranted before considering more advanced biological models.

## 4. Materials and Methods

### 4.1. Chemistry

#### 4.1.1. Reagents and Instruments

All reagents used and the 60 F254 silica plates were obtained from Merck (Darmstadt, Germany). The reagents were used without additional purification. Melting points were determined using a Biobase melting point analyzer (Biobase Group, Jinan, China). The ^1^H and ^13^C NMR experiments were recorded on a Bruker Avance NEO 400 MHz Spectrometer, equipped with a 5 mm QNP direct detection probe and z−gradients. The spectra were recorded at room temperature in DMSO-d_6_ using default parameter sets, as provided by Bruker and were referenced on the residual peak of the solvent (^1^H: 2.50 ppm; ^13^C: 39.5 ppm). The NMR spectra were first recorded in DMSO-d_6_, then, in each NMR tube containing the solutions, a drop of trifluoroacetic acid (TFA) was added; the solutions were vortexed for 5 min at 500 rpm, and then the NMR experiments were recorded again. Fourier-transform infrared spectroscopy (FTIR) analysis was achieved using KBr pellets on a Shimadzu IR Affinity-1S spectrometer (Kyoto, Japan, 400–4000 cm^−1^ range and a 4 cm^−1^ resolution). High performance liquid chromatography (HPLC/LC) coupled with mass spectrometry (MS) experiments were conducted on a 6120 LC-MS analytical system from Agilent (Santa Clara, CA, USA) consisting of 1260 Infinity HPLC equipped with G1322A degasser, G1311B quaternary pump, G1316A column thermostat, G1365C MWD detector, G7129A autosampler coupled with a Quadrupolar (Q) mass spectrometer equipped with electrospray ionization source (ESI). Screening of **MG-1**–**4** compounds was conducted by LC-MS, on a reverse phase Zorbax Eclipse Plus C18 column (3.0 × 100 mm × 3.5 µm). The 4 compounds were screened in isocratic elution with a mobile phase that consists of a mixture of 10% 1 mM ammonium formate solution and 90% methanol. The elution of all components was achieved at a flow rate of 0.5 mL/min, injection volume 5 µL and column temperature of 40 °C. UV detection of compounds was conducted at 250 nm. MS detection was achieved by electrospray ionization (ESI) in the positive ion mode using single ion monitoring (SIM). MS parameters: capillary voltage 3000 V, dry gas flow 12 L/min at 350 °C, nebulizer pressure 35 psig and fragmentor at 70. The *m*/*z* scale of the mass spectrum was calibrated by use of an external calibration standard ESI Tuning Mix from Agilent (Santa Clara, CA, USA). All obtained spectra are available in the Supplementary Material file associated with this manuscript (Figures S1–S13).

#### 4.1.2. Synthesis of 1*H*-Benzotriazole (BzTz) and 1-(2-Bromoethyl)-benzotriazole (BzTzBr)

The synthesis of 1*H*-benzotriazole was achieved by the diazotization reaction of *o*-phenylenediamine in acetic acid medium, according to a previously reported method [27]. A quantity of 5 g of *o*-phenylenediamine (46.24 mmol) and NaNO_2_ (3.51 g, 101.72 mmol) were dissolved, under stirring, in 70 mL of glacial CH_3_COOH in a round-bottomed flask (200 mL), equipped with a magnetic stirrer. After stirring at room temperature for 1 h, the mixture was diluted with water and extracted with ethyl acetate. The combined organic layers were dried over anhydrous Na_2_SO_4_, and after evaporation of the solvent the crude product was obtained as a white solid (5.15 g, 43.23 mmol, η = 93%).

The synthesis of 1-(2-bromoethyl)-benzotriazole (BzTzBr) was carried out after a slightly modified previously published method [28]. BzTz (3 g, 25.18 mmol) was dissolved in 5 mL DMF, followed by the addition of potassium carbonate (10.44 g, 75.5 mmol). The reaction mixture was cooled down using an ice bath, and a previously prepared solution of 1,2-dibromoethane (21.8 mL, 251.8 mmol) in 5 mL DMF was added in one portion under strong stirring. The mixture was allowed to react at room temperature for 24 h. TLC confirmed complete consumption of the starting material, and the reaction mixture was poured into an ice bath and extracted with ethyl acetate several times. Collected organic phases were washed with water, brine, and dried over Na_2_SO_4_. Solvent was removed by rotary evaporation, and the resulting yellow liquid residue was triturated with hexanes, resulting in the slow precipitation of the 1-(2-bromoethyl)benzotriazole (1.55 g, 6.85 mmol, η = 27%) as a white solid, which was used without further purification in the next step.

#### 4.1.3. General Synthesis Procedure for Compounds **MG-1**–**4**

The synthesis and characterization of 5-R-1,2,4-triazole-3-thiols (compounds **1a**–**1d**) used for the synthesis of compounds **MG-1**–**4** were previously reported [22].

**Figure 17:**
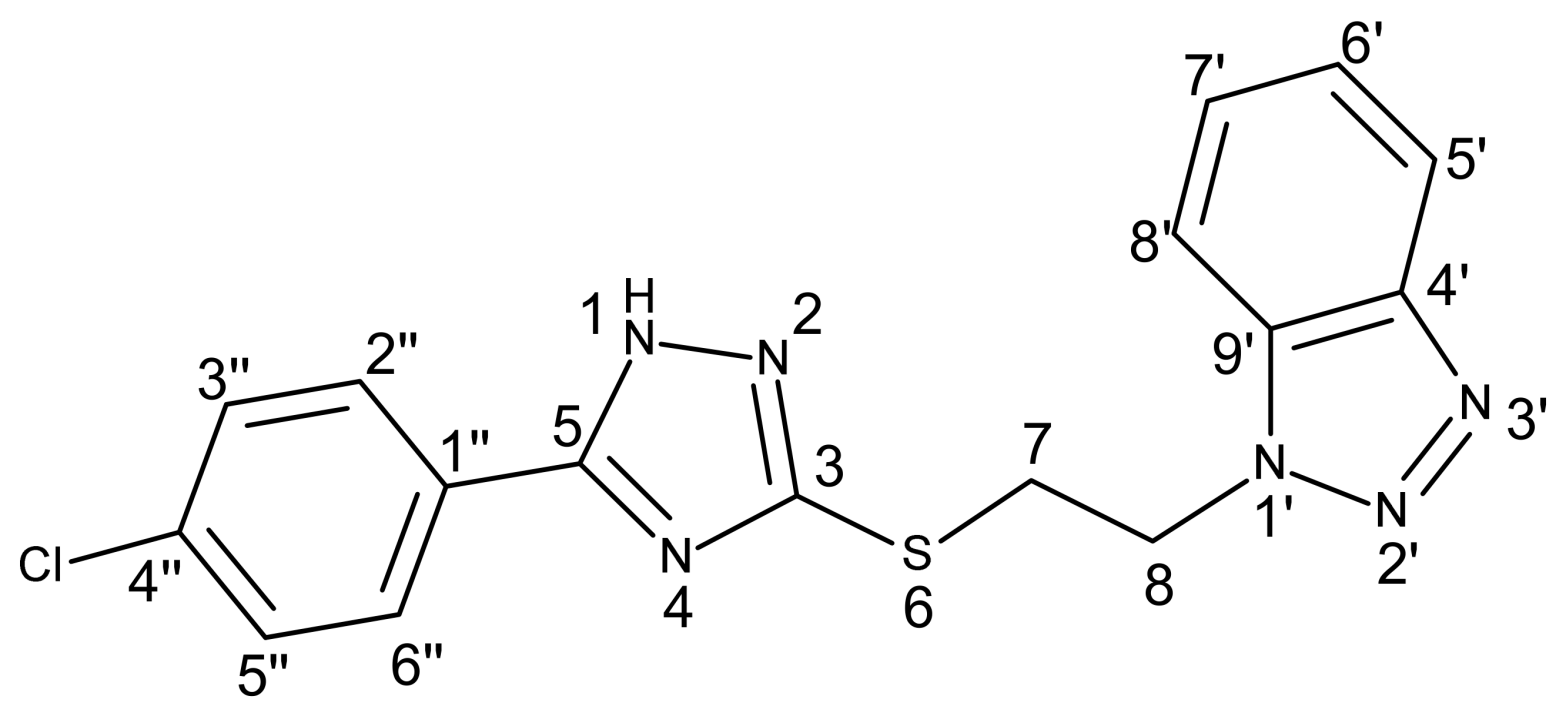
1-[2-[[5-(4-chlorophenyl)-1*H*-1,2,4-triazol-3-yl]sulfanyl]ethyl]benzotriazole (**MG-1**).

**Figure 18:**
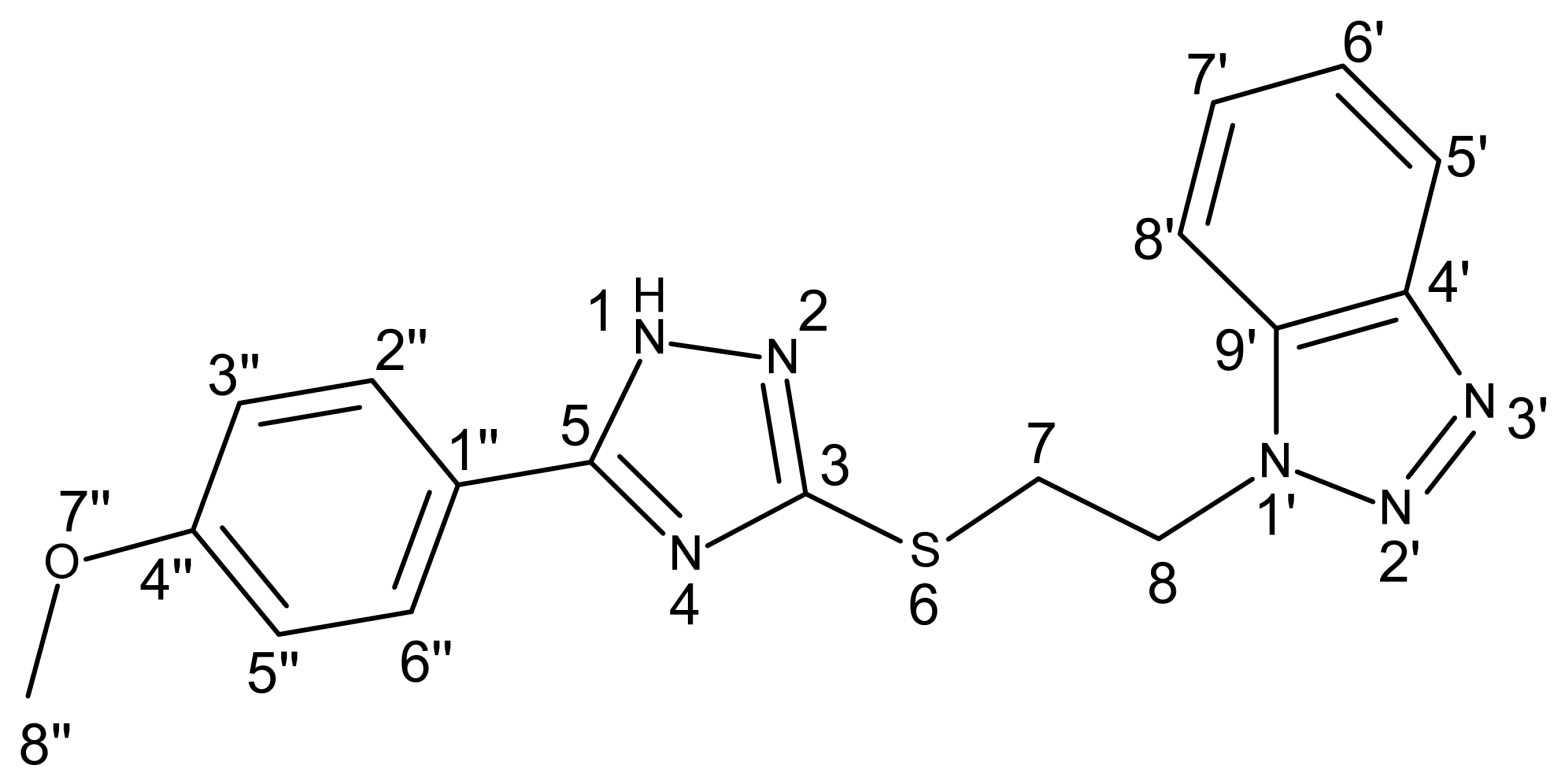
1-[2-[[5-(4-methoxyphenyl)-1*H*-1,2,4-triazol-3-yl]sulfanyl]ethyl]benzotriazole (**MG-2**).

**Figure 19:**
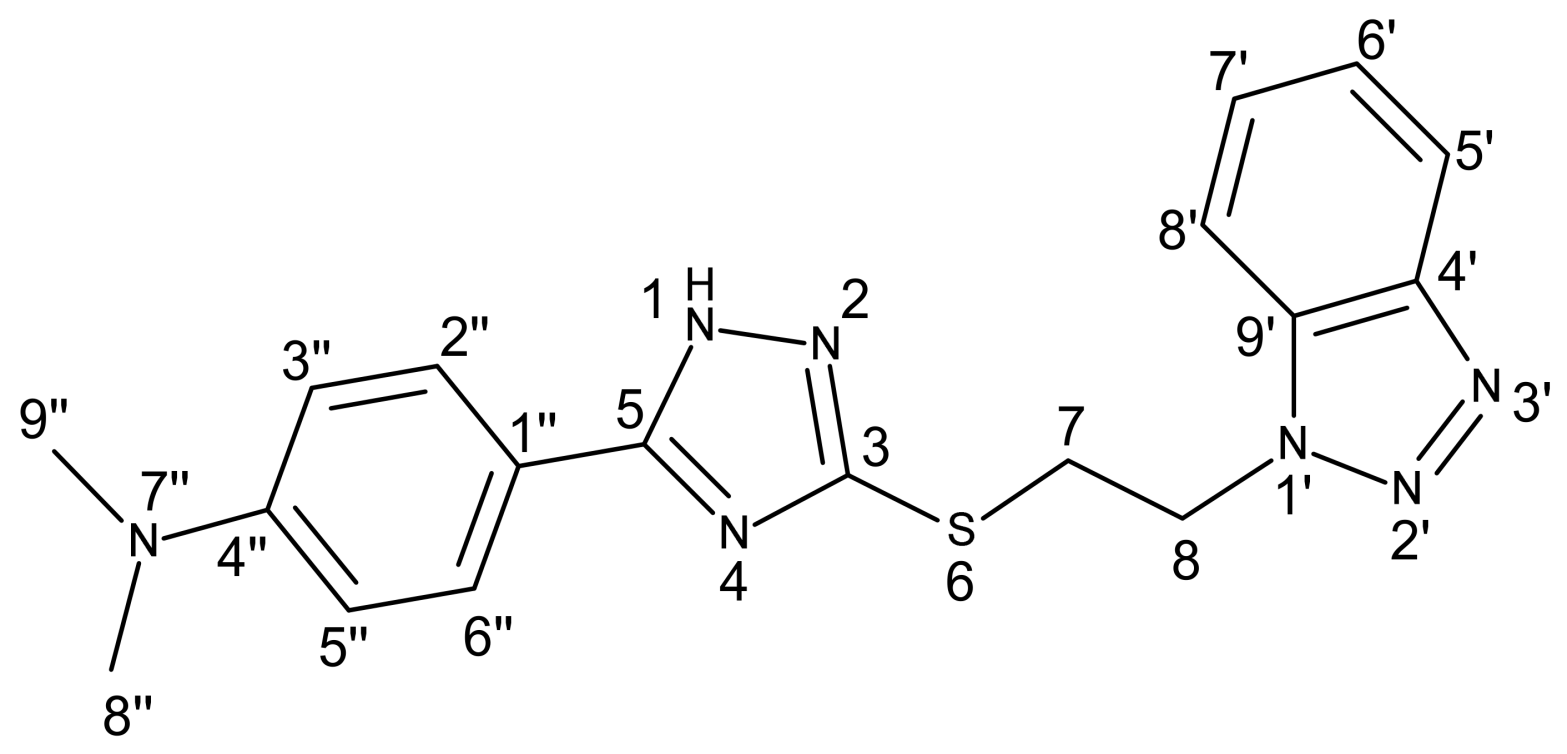
4-[3-[2-(benzotriazol-1-yl)ethylsulfanyl]-1*H*-1,2,4-triazol-5-yl]-N,N-dimethyl-aniline (**MG-3**).

**Figure 20:**
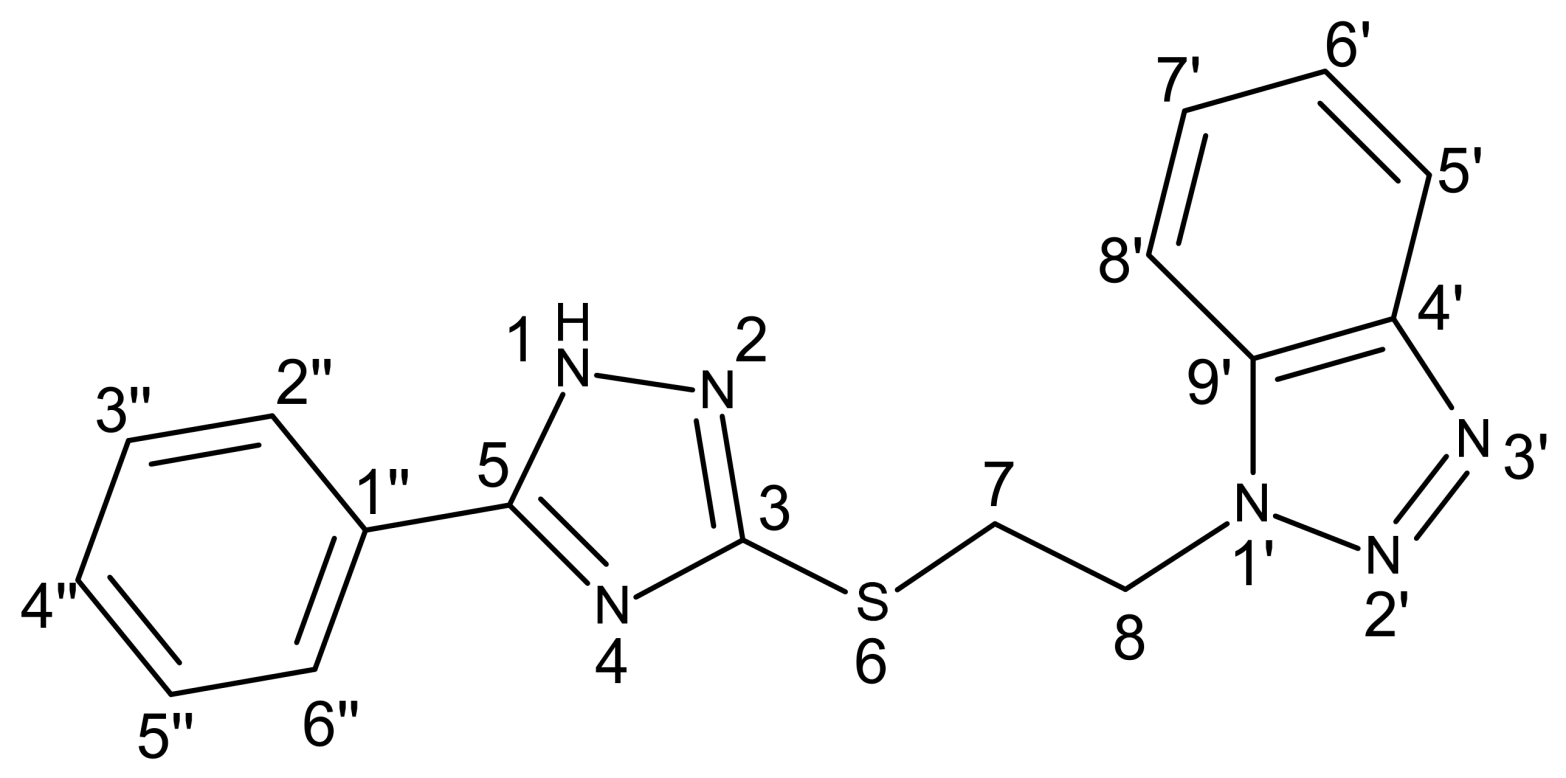
1-[2-[(5-phenyl-1*H*-1,2,4-triazol-3-yl)sulfanyl]ethyl]benzotriazole (**MG-4**).

To obtain compounds **MG-1**–**4** (Figure 17, Figure 18, Figure 19 and Figure 20), each triazole reacted with the intermediate BzTzBr to yield 1,2,4-triazole-benzotriazole hybrids. For this purpose, 1.05 mmol 5-R-1*H*-1,2,4-triazole-3-thiol was dissolved in 10 mL acetonitrile, followed by the addition in one portion of potassium carbonate (365 mg, 2.64 mmol); 10 mL of a previously prepared acetonitrile solution of BzTzBr (200 mg, 0.88 mmol) was added dropwise into the reaction mixture, allowing it to react overnight. After TLC analysis showed complete consumption of the starting material, the acetonitrile was removed by rotary evaporation, and the resulting solid residue was dissolved in ethyl acetate, washed with distilled water and brine, dried over anhydrous Na_2_SO_4_ and the organic solvent was removed to yield a white solid residue. Compounds were purified by column chromatography (SiO_2_/50% ethyl acetate in hexane).

White solid, yield 36%, m.p. 170–175 °C, R*_f_* = 0.28 (Ethyl acetate: hexane, 1:1); ^1^H NMR (DMSO-d_6_ + TFA, 400.13 MHz, δ, ppm): 8.32 (d, J = 8.3 Hz, 1H, H5′), 7.93–7.89 (m, 3H, H2″. H6″, H8′), 7.58 (d, J = 8.5 Hz, 2H, H3″, H5″), 7.53 (t, J = 7.6 Hz, 1H, H7′), 7.37 (t, J = 7.6 Hz, 1H, H6′), 5.11 (t, J = 6.6 Hz, 2H, H8), 3.75 (t, J = 6.6 Hz, 2H, H7). ^13^C NMR (DMSO-d_6_ + TFA, 100.13 MHz, δ, ppm): 156.8 (C5), 155.9 (C3), 145.1 (C4′), 134.5 (C4″), 133.0 (C9′), 129.0 (C1″, C3″, C5″), 127.7 (C2″, C6:), 127.2 (C7′), 123.9 (C6′), 119.1 (C5′), 110.6 (C8′), 47.2 (C8), 31.1 (C7); FTIR (KBr, cm^−1^) relevant peaks: 3073–3140 (aromatic C–H and N–H stretching); 2924, 2870 (aliphatic CH_2_ stretching); 1610, 1579 (C=N stretching of triazole and benzotriazole rings, aromatic C=C stretching); 1393, 1354, 1329, 1269, 1217, 1157, 1134 (C–N and C–S stretching); 1089, 1020 (C–N stretching); 835, 794, 773, 739 (aromatic C–H out-of-plane bending); 675, 662 (C–Cl stretching). ESI-MS: **MG-1** identified as [M + H]^+^ at *m*/*z* = 357.2 and Rt = 1.217 min.

White solid, yield 35.6%, m.p. 139–143 °C, R*_f_* = 0.17 (Ethyl acetate: hexane, 1:1); ^1^H NMR (DMSO-d_6_ + TFA, 400.13 MHz, δ, ppm): 8.01 (d, J = 8.3 Hz, 1H, H5′), 7.93–7.87 (m, 3H, H3″. H5″, H8′), 7.54 (t, J = 7.6 Hz, 1H, H7′), 7.38 (t, J = 7.6 Hz, 1H, H6′). 7.08 (d, J = 8.6 Hz, 2H, H2″, H6″), 5.10 (t, J = 6.6 Hz, 2H, H8), 3.82 (s, 3H, H8″), 3.71(t, J = 6.6 Hz, 2H, H7), ^13^C NMR (DMSO-d_6_ + TFA, 100.13 MHz, δ, ppm): 160.8 (C4″) 156.8 (C5), 156.5 (C3), 145.2 (C4′), 133.1 (C9′), 127.8 (C1″), 127.7 (C3″, C5″), 127.3 (C7′) 124.0 (C6′), 119.1 (C5′), 114.5 (C2″, C6″), 110.7 (C8′), 55.4 (C8″), 47.2 (C8), 30.9 (C7). FTIR (KBr, cm^−1^) relevant peaks: 3000–3100 (aromatic C–H and N–H stretching); 2934, 2868 (aliphatic C–H stretching); 1614, 1579 (C=N stretching of the triazole and benzotriazole rings, aromatic C=C stretching); 1265, 1225, 1094, 1026 (C–O/C–O–C stretching of the methoxy group); 839, 782, 768, 745 (aromatic C–H out-of-plane bending). ESI-MS: **MG-2** identified as [M + H]^+^ at *m*/*z* = 353.2 and Rt = 1.134 min.

White solid, yield 51.4%, m.p. 156–160 °C, R*_f_* = 0.17 (Ethyl acetate: hexane, 1:1); ^1^H NMR (DMSO-d_6_ + TFA, 400.13 MHz, δ, ppm): 8.02 (d, J = 8.3 Hz, 1H, H5′), 7.93 (d, J = 8.3 Hz, 1H, H8′), 7.80 (d, J = 8.7 Hz, 2H, H2″. H6″), 7.54 (t, J = 7.6 Hz, 1H, H7′), 7.39 (t, J = 7.6 Hz, 1H, H6′), 6.89 (d, J = 8.7 Hz, 2H, H3″, H5″), 5.10 (t, J = 6.7 Hz, 2H, H8), 3.70 (t, J = 6.7 Hz, 2H, H7), 3.00 (s, 6H, H8″, H9″), ^13^C NMR (DMSO-d_6_ + TFA, 100.13 MHz, δ, ppm): 156.6 (C5), 156.5 (C3), 151.0 (C4″), 145.2 (C4′), 133.0 (C9′), 127.3 (C2″, C6″), 127.2 (C7′), 124.0 (C6′), 119.1 (C5′), 114.9 (C1″), 112.6 (C3″, C5″), 110.7 (C8′), 47.2 (C8), 40.2 (C8″, C9″), 30.8 (C7). FTIR (KBr, cm^−1^) relevant peaks: 3065–3000 (aromatic C–H and N–H stretching); 2940, 2886, 2816 (aliphatic C–H stretching); 1614, 1557 (C=N stretching of the triazole and benzotriazole rings, aromatic C=C stretching); 1315, 1290, 1273, 1254 (C–N stretching of the azole rings and N(CH_3_)_2_ group); 1199, 1163, 1128, 1113 (C–N stretching); 1094, 1001 (C–N stretching and aromatic in-plane C–H deformation); 820, 781, 768, 746 (aromatic C–H out-of-plane bending). ESI-MS: **MG-3** identified as [M + H]^+^ at *m*/*z* = 366.3 and Rt = 1.155 min.

White solid, yield 21%, m.p. 145–150 °C, R*_f_* = 0.28 (Ethyl acetate: hexane, 1:1); ^1^H NMR (DMSO-d_6_ + TFA, 400.13 MHz, δ, ppm): 7.99 (d, J = 8.3 Hz, 1H, H5′), 7.95–7.90 (m, 3H, H2″, H6″, H8′), 7.54–7.47 (m, 4H, H3″-5″, H7′), 7.37 (t, J = 7.6 Hz, 1H, H6′), 5.11 (t, J = 6.7 Hz, 2H, H8), 3.74 (t, J = 6.5 Hz, 2H, H7), ^13^C NMR (DMSO-d_6_ + TFA, 100.13 MHz, δ, ppm): 157.2 (C5), 156.6 (C3), 145.3 (C4′), 133.2 (C9′), 130.2 (C4″), 129.1 (C3″, C5″), 128.6 (C1″), 127.3 (C7′), 126.2 (C2″, C6″), 124.1 (C6′), 119.2 (C5′), 110.7 (C8′), 47.3 (C8), 31.1 (C7). FTIR (KBr, cm^−1^) relevant peaks: 3140–3003 (aromatic C–H and N–H stretching); 2929, 2864 (aliphatic C–H stretching); 1632, 1578 (C=N stretching of the triazole and benzotriazole rings, aromatic C=C stretching); 1333, 1275, 1240, 1217 (C–N and C–S stretching); 779, 748, 721, 691 (aromatic C–H out-of-plane bending). ESI-MS: **MG-4** identified as [M + H]^+^ at *m*/*z* = 323.2 and Rt = 1.146 min.

### 4.2. Biological Assessment

#### 4.2.1. Cell Culture

In this study, four cell lines were used: one non-tumoral human keratinocyte cell line (HaCaT) and three human cancer cell lines (A375, A549, HT-29). The HaCaT cell line was purchased from CLS Cell Lines Service GmbH (Eppelheim, Germany), while A375 (melanoma), A549 (non-small cell lung cancer), and HT-29 (colorectal adenocarcinoma) were purchased from ATCC (Łomianki, Poland). The HaCaT and A375 cell lines were cultured in Dulbecco’s Modified Eagle Medium (DMEM), while A549 was cultured in Dulbecco’s Modified Eagle Medium (DMEM)/Nutrient Mixture F-12 Ham and HT-29 cells in McCoy’s 5A medium. In addition, all media were supplemented with 10% fetal bovine serum (FBS) and 1% penicillin/streptomycin and the cells were kept at 37 °C in a humidified atmosphere containing 5% CO_2_.

#### 4.2.2. Cell Viability Assay

To evaluate the effects of benzotriazole (BzTz) and its hybrids on cell viability, the four cell lines—HaCaT, HT-29, A375, and A549—were seeded at a concentration of 1 × 10^4^ cells per well in 96-well plates. The cells were allowed to adhere and grow until they reached 85–90% confluence, after which they were treated with fresh medium containing benzotriazole (BzTz), its final hybrids (**MG-1**, **MG-2**, **MG-3**, **MG-4**), and the corresponding synthetic intermediates (**1a**–**d**). All compounds were tested at concentrations of 10, 25, 50, 75, and 100 μM, obtained by diluting a 20 mM stock solution, which was prepared by dissolving each test compound in 500 μL of dimethylsulfoxide (DMSO). After treatment, the cells were incubated for 48 h, then 20 μL of 0.01% Alamar Blue solution was added to each well, and the cells were further incubated for 3 h, according to the manufacturer’s protocol. Absorbance was measured at 570 nm using a microplate reader (xMark™ Microplate, Bio-Rad Laboratories, Hercules, CA, USA). Cell viability was expressed as a percentage of the control value (untreated cells), which was 100%, and the experiments were performed in triplicate. Under experimental conditions similar to those used for testing the final compounds, synthetic intermediates were also tested. Consequently, the results of these tests are presented in the Supplementary Material (Table S1 and Figures S14–S17), as they did not exhibit any biologically relevant cytotoxic effects within the tested concentration range.

#### 4.2.3. Cell Morphology Assay

HaCaT, A375, A549 and HT-29 cells were seeded in 12-well plates at an initial density of 2 × 10^5^ cells per well. Upon reaching approximately 80–85% confluence, the culture medium was replaced with fresh medium containing benzotriazole (BzTz) and its final hybrids (**MG-1**, **MG-2**, **MG-3**, **MG-4**). Treatment concentrations were selected based on the IC_50_ values derived from the cell viability data for each cell line whenever available; for compounds that did not reach 50% inhibition within the investigated concentration range, the highest tested concentration (100 μM) was used. The cells were incubated for 48 h under standard culture conditions. Following treatment, cellular morphology was examined using the EVOS™ M5000 Imaging System equipped with a high-sensitivity CMOS camera (Thermo Fisher Scientific, Inc., Waltham, MA, USA). Morphological observations were used as qualitative support for the viability data.

#### 4.2.4. Immunofluorescence Assay—Assessment of Nuclear and Cytoskeletal Alterations

The immunofluorescence assay was performed on the four cell lines (HaCaT, A375, A549, HT-29), following a 48-h treatment with benzotriazole (BzTz) and its final hybrids **MG-1**, **MG-2**, **MG-3**, and **MG-4** at concentrations corresponding to the IC_50_ values obtained from cell viability data, whenever these were available; if IC_50_ values could not be determined, the highest tested concentration, 100 μM, was used. Staurosporine at 5 μM was used as a positive control and was added to some wells. Before treatment, each cell line was cultured in 12-well plates at a concentration of 2 × 10^5^ cells/well and allowed to proliferate until 80–85% confluence was reached. After treatment, the cells were fixed with 4% paraformaldehyde for 15 min, permeabilized with 0.25% Triton™ X-100 for 10 min, and subsequently blocked with 5% bovine serum albumin (BSA) for one hour at room temperature. To visualize the changes, a mouse monoclonal antibody against β-actin (2 µg/mL, MA5-15739, Thermo Fisher Scientific, USA) diluted in 0.1% BSA for three hours at room temperature, followed by labeling with the Alexa Fluor™ 488 anti-mouse goat secondary antibody (1:2000 dilution, A28175, Thermo Fisher Scientific, USA) for 45 min. For the final step, we added the prepared Hoechst 33258 solution and let it sit for 5 min in the dark. Using the EVOS™ M5000 imaging system, equipped with a high-sensitivity CMOS camera (Thermo Fisher Scientific, Inc., Waltham, MA, USA), we were able to detect changes in fluorescence.

### 4.3. Statistical Analysis

Statistical analysis was performed using one-way ANOVA (GraphPad Prism version 6.0.0, GraphPad Software, San Diego, CA, USA) for comparisons performed separately within each experimental setting. To evaluate the differences between the biological control, benzotriazole, and each benzotriazole derivative, the Bonferroni multiple comparison test was used, while to additionally explore the impact of derivatization relative to the parent scaffold, the Dunnett multiple comparison test was used. Since benzotriazole did not reach an IC_50_ within the investigated concentration range, the response obtained at 100 μM was selected as the reference condition for the parent scaffold. Thus, the effects obtained for the benzotriazole hybrids at each tested concentration (10, 25, 50, 75, 100 μM) were compared with the effect of BzTz at 100 μM to determine whether derivatization produced a stronger response than the maximum effect observed for the parent compound under the tested conditions. Differences were considered statistically significant at *p* < 0.05. Statistical significance relative to the biological control is indicated by asterisks (* *p* < 0.05, ** *p* < 0.01, *** *p* < 0.001), while significance between compounds and benzotriazole is indicated by hash symbols (# *p* < 0.05, ## *p* < 0.01, ### *p* < 0.001) relative to BzTz tested at 100 μM.

### 4.4. Molecular Docking

Molecular docking studies were performed using AutoDock Vina (v1.1.2) implemented in PyRx (version 0.8) [29]. The 3D structures of the selected protein targets were obtained from the Protein Data Bank (PDB) [30]. Prior to docking, the co-crystallized ligands and water molecules were removed using BIOVIA Discovery Studio Visualizer v20.1.0.19295 (Dassault Systèmes, Paris, France). The resulting protein structures were imported into PyRx and prepared as macromolecules using the available integrated AutoDock, followed by conversion to the PDBQT format.

The PDB identifiers, corresponding co-crystallized ligands, and the coordinates and dimensions of the docking grid boxes are summarized in Table 4. For each target, the grid box was positioned to encompass the binding pocket defined by the co-crystallized ligand and the surrounding binding-site region.

The structures of compounds **MG-1**–**MG-4** were drawn using BIOVIA Draw and imported into PyRx. Ligand geometries were energy-minimized using Open Babel with the Universal Force Field (UFF) and subsequently converted to the PDBQT format. Docking calculations were performed with an exhaustiveness value of 8. For each ligand–protein pair, multiple binding poses were generated and ranked according to the AutoDock Vina predicted binding affinity, expressed in kcal/mol. The pose with the lowest predicted binding energy was retained for further analysis.

The co-crystallized ligand of each target was redocked using the same protocol to assess the ability of the docking procedure to reproduce the experimentally observed binding orientation. The redocked poses were compared with the corresponding crystallographic ligand conformations, and only docking protocols for which the root-mean-square deviation (RMSD) between the experimental and redocked ligand poses was below 2.0 Å were retained for further analysis.

### 4.5. Molecular Dynamics Simulation and MM-GBSA Analysis

The dynamic behaviour of the 3DV3–**MG-1** and 3DV3–**MG-3** complexes was investigated by molecular dynamics (MD) simulations performed with Desmond, available within the Schrödinger suite (Schrödinger Release 2025-1, Schrödinger, LLC, New York, NY, USA). The protein–ligand complexes were prepared using the System Builder module and placed in an orthorhombic simulation box containing SPC water molecules, maintaining a 10 Å buffer between the solute and the box boundaries. The net charge of each system was neutralized with appropriate counterions, followed by the addition of NaCl to a concentration of 0.15 M. Before the production MD simulations, energy minimization and equilibration were performed for both systems according to the standard Desmond protocol. Each complex was then simulated for 100 ns in the NPT ensemble, maintaining the temperature at 300 K and the pressure at 1.01325 bar. Coordinates were saved every 100 ps, providing approximately 1000 trajectory frames for each simulation. The resulting trajectories were examined using the Simulation Interaction Diagram tools implemented in Maestro. Structural stability and flexibility were characterized using root-mean-square deviation (RMSD) and root-mean-square fluctuation (RMSF) analyses, while the persistence and evolution of protein–ligand contacts were monitored throughout the trajectories.

The binding energetics of **MG-1** and **MG-3** toward 3DV3 were further assessed using Prime MM-GBSA implemented in Schrödinger (Schrödinger Release 2025-1, Schrödinger, LLC, New York, NY, USA). Calculations were carried out using the OPLS4 force field in combination with the VSGB implicit solvent model. During refinement, each ligand and protein residues located within 5 Å of the ligand were subjected to local minimization, whereas the remaining receptor atoms were maintained fixed. The resulting binding free-energy estimates (ΔG_bind) were evaluated together with their principal energetic contributions, including van der Waals, Coulombic, hydrogen-bonding, lipophilic, packing, and generalized Born solvation terms, as well as ligand efficiency (LE).

## 5. Conclusions

This study reports the synthesis, structural characterization, and in vitro evaluation of the antitumor potential of a small series of four new benzotriazole–1,2,4-triazole hybrids (**MG-1**–**MG-4**). Regarding the evaluation of antitumor activity, we observed a reduction in metabolic viability that was dependent on the cell line and the derivative. Thus, the most active compounds in the series were compounds **MG-1** and **MG-3**. **MG-1** was the most active compound in the series against A375 (IC_50_ = 75.43 ± 2.77 μM), while **MG-3** was the most active against the other two tumor lines tested, namely A549 (IC_50_ = 56.78 ± 2.57 μM) and HT-29 (IC_50_ = 69.19 ± 3.97 μM). **MG-2** achieved an IC_50_ only when treating the A549 line, whereas **MG-4** failed to achieve a measurable IC_50_ within the tested concentration range. We could interpret the antitumor activity results as a preliminary relationship between the terminal aryl substitution and the biological response, but the remaining number of compounds evaluated prevents us from establishing a clear structure–activity relationship. The fact that no IC_50_ was obtained on keratinocytes up to 100 μM, and that the reduction in cell viability was moderate when testing the highest concentration (100 μM), suggests only a modest difference in sensitivity between tumor and nontumor cells and not a demonstrated selectivity toward tumor cells. The changes identified by immunofluorescence (nuclear and cytoskeletal) merely confirm the results of the Alamar Blue viability assay, particularly in the case of **MG-1** and **MG-3**, but do not establish a specific mechanism of cell death. However, to confirm specific mechanisms—whether or not they involve apoptosis—additional functional assays are undoubtedly necessary.

Overall, **MG-1** and **MG-3** can be considered the most promising lead compounds in this limited series, although their antitumor activity remains moderate. Future studies should focus on expanding the chemical series, improving potency and selectivity, confirming the effects on additional tumor and nontumor cell models, and elucidating the underlying mechanisms before proceeding to in vivo evaluation.

## Figures and Tables

**Table 1:** Calculated IC_50_ values (μM) for BzTz, **MG-1**, **MG-2**, **MG-3**, and **MG-4** on the HaCaT, A375, A549, and HT-29 cell lines at 48 h. For compounds that did not reach 50% inhibition within the tested concentration range, IC_50_ values are reported as >100 μM. IC_50_ values obtained after 48 h of treatment (µM).

Compounds	HaCaT	A375	A549	HT-29
**BzTz**	>100	>100	>100	>100
**MG-1**	>100	75.43 ± 2.77	77.15 ± 1.32	96.28 ± 1.72
**MG-2**	>100	>100	90.20 ± 5.09	>100
**MG-3**	>100	78.97 ± 3.08	56.78 ± 2.57	69.19 ± 3.97
**MG-4**	>100	>100	>100	>100

**Table 2:** Predicted binding affinities of **MG-1**–**MG-4** and the corresponding native ligands (NL) against the investigated protein targets. Docking scores are expressed in kcal/mol.

Protein	PDB ID	NL	MG-1	MG-2	MG-3	MG-4
**EGFR**	1XKK	−10.8	−9.3	−9.0	−8.7	−9.3
**BRAF^V600E**	3OG7	−11.2	−8.4	−8.6	−8.4	−8.5
**MEK1**	3DV3	**−9.4**	**−9.4**	−9.0	−9.2	−9.2
**PI3Kα**	6GVF	−8.8	−8.3	−8.2	−8.0	−8.2
**AKT1/PKBα**	4GV1	−9.4	−9.1	−8.6	−8.7	−8.8
**FAK/PTK2**	6I8Z	−11.3	−7.9	−7.8	−7.8	−8.3
**BCL-2**	6O0K	−12.3	−8.0	−7.9	−8.2	−8.1
**BCL-xL**	2YXJ	−10.9	−8.2	−8.1	−8.3	−8.0

**Table 3:** Prime MM-GBSA binding free energies and principal energetic contributions calculated for the MEK1–**MG-1** and MEK1–**MG-3** complexes. Energy values are expressed in kcal/mol. Negative values indicate favorable energetic contributions, whereas positive values indicate energetic penalties.

MM-GBSA Parameter	MEK1–MG-1 (kcal/mol)	MEK1–MG-3 (kcal/mol)
ΔG_bind total	−54.83	−40.70
van der Waals	−45.53	−50.90
Coulomb	−16.66	−23.30
H-bond	−0.32	−1.66
Lipophilic	−22.96	−10.89
Packing	−0.91	−0.06
Solv_GB	+26.73	+39.45
Ligand strain energy	+6.06	+6.94

**Table 4:** Protein targets, PDB structures, docking grid parameters, and corresponding co-crystallized ligands used in the molecular docking study.

Protein (PDB ID)	Grid Box Center (x, y, z), Å	Grid Box Size (x, y, z), Å	Native Ligand
**EGFR (1XKK)**	16.2665, 32.9271, 36.2856	19.7144, 19.7144, 19.6332	Lapatinib
**BRAF^V600E (3OG7)**	2.8424, −1.6282, −18.9946	18.4660, 18.4660, 18.4660	Vemurafenib (PLX4032)
**MEK1 (3DV3)**	39.5681, −12.6840, −1.1443	9.0338, 14.7621, 11.8738	PF-04622664
**PI3Kα (6GVF)**	−17.0667, 146.8764, 28.6252	12.6709, 15.0364, 12.6709	Sapanisertib
**AKT1/PKBα (4GV1)**	−20.3313, 6.8247, 10.8312	18.4660, 18.4660, 18.4660	AZD5363 (Capivasertib)
**FAK/PTK2 (6I8Z)**	7.3297, 27.7992, 31.2637	18.4660, 18.9297, 18.4660	BI-4464
**BCL-2 (6O0K)**	−14.3014, 0.2388, −9.9689	18.4659, 15.4559, 23.5720	Venetoclax
**BCL-xL (2YXJ)**	−9.6142, −15.0938, 9.5863	22.8015, 25.0000, 12.3139	ABT-737

## Data Availability

The original contributions presented in this study are included in the article and its Supplementary Materials. Further inquiries can be directed to the corresponding author.

## Supplementary Materials

The following supporting information can be downloaded at https://www.mdpi.com/article/10.3390/ph19101591/s1, Figure S1: ^1^H NMR for **MG-1** in DMSO-d_6_ (bottom) and DMSO-d_6_ + TFA (up).; Figure S2: ^13^C NMR for **MG-1** in DMSO-d_6_ (bottom) and DMSO-d_6_ + TFA (up); Figure S3: FTIR spectra of compound **MG-1**; Figure S4: ^1^H NMR for **MG-2** in DMSO-d_6_ (bottom) and DMSO-d_6_ + TFA (up); Figure S5: ^13^C NMR for **MG-2** in DMSO-d_6_ (bottom) and DMSO-d_6_ + TFA (up); Figure S6: FTIR spectra of compound **MG-2**; Figure S7: ^1^H NMR for **MG-3** in DMSO-d_6_ (bottom) and DMSO-d_6_ + TFA (up); Figure S8: ^13^C NMR for **MG-3** in DMSO-d_6_ (bottom) and DMSO-d_6_ + TFA (up); Figure S9: FTIR spectra of compound **MG-3**; Figure S10: ^1^H NMR for **MG-4** in DMSO-d_6_ (bottom) and DMSO-d_6_ + TFA (up); Figure S11: ^13^C NMR for **MG-4** in DMSO-d_6_ (bottom) and DMSO-d_6_ + TFA (up); Figure S12: FTIR spectra of compound **MG-4;** Figure S13: LC–MS spectra of compounds **MG-1**–**MG-4;** Figure S14: Effects of BzTz and synthetic intermediates **1a**–**1d** on HaCaT cell viability after 48 h of treatment; Figure S15: Effects of BzTz and synthetic intermediates **1a**–**1d** on A375 cell viability after 48 h of treatment; Figure S16: Effects of BzTz and synthetic intermediates **1a**–**1d** on A549 cell viability after 48 h of treatment; Figure S17: Effects of BzTz and synthetic intermediates **1a**–**1d** on HT-29 cell viability after 48 h of treatment; Table S1: summary of cell viability values at 100 μM for BzTz and synthetic intermediates **1a**–**1d**; Table S2: microbial species tested; Table S3: antimicrobial activity of BzTz, synthetic intermediates **1a**–**1d**, and final benzotriazole–1,2,4-triazole hybrids **MG-1**–**MG-4** at 500 μg/mL. References [31,32,33] are cited in the Supplementary Materials.
