# Supplementary material for: In Vitro and In Silico Evaluation of the Antitumor Potential of New Benzotriazole–1,2,4-Triazole Hybrids
Source: Pharmaceuticals (Basel). 2026 Oct 8;19(10):1591. doi: 10.3390/ph19101591 (PMC13708708; doi:10.3390/ph19101591)
Supplement: Supplementary file 1 [file pharmaceuticals-19-01591-s001.zip › pharmaceuticals-4563656-supplementary.pdf]

# Supplementary File

## 1. Spectral data for compounds MG-1 – MG-4

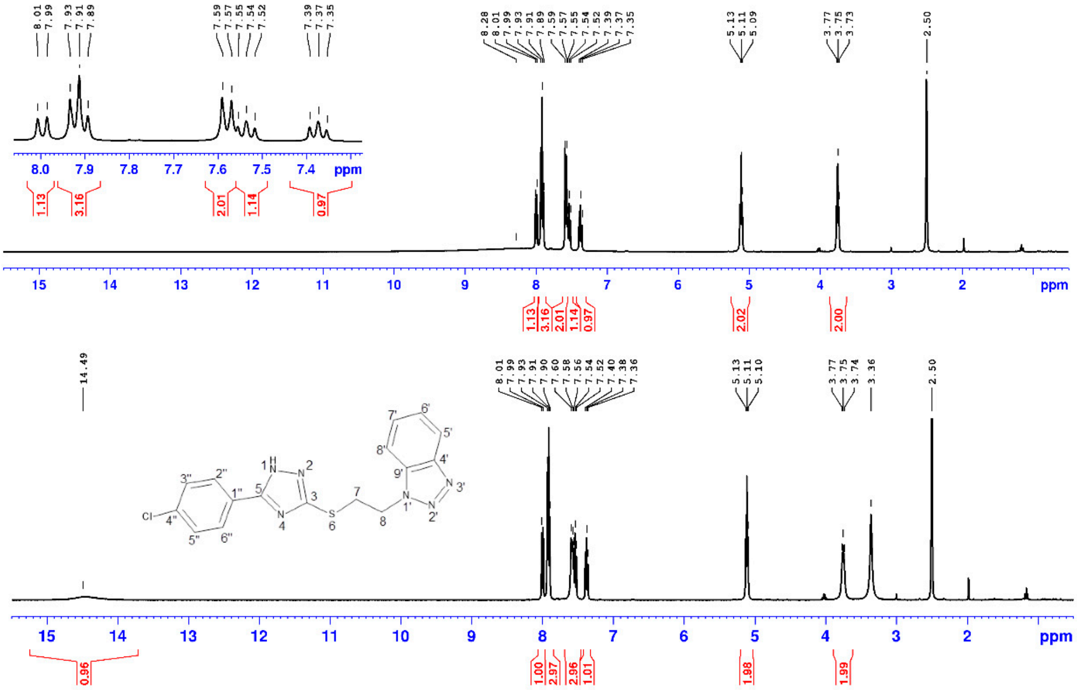


**Figure S1.** $^1$H NMR for **MG-1** in DMSO-$d_6$ (bottom) and DMSO-$d_6$ +TFA (up).

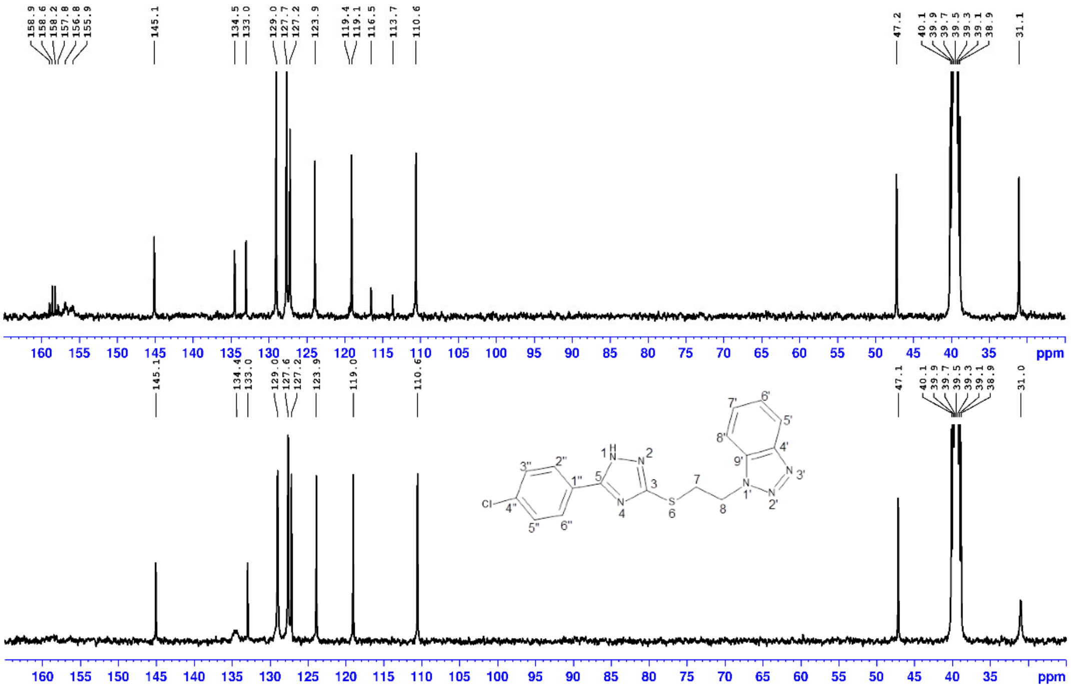

**Figure S2.** $^{13}C$ NMR for **MG-1** in DMSO-$d_6$ (bottom) and DMSO-$d_6$ +TFA (up).

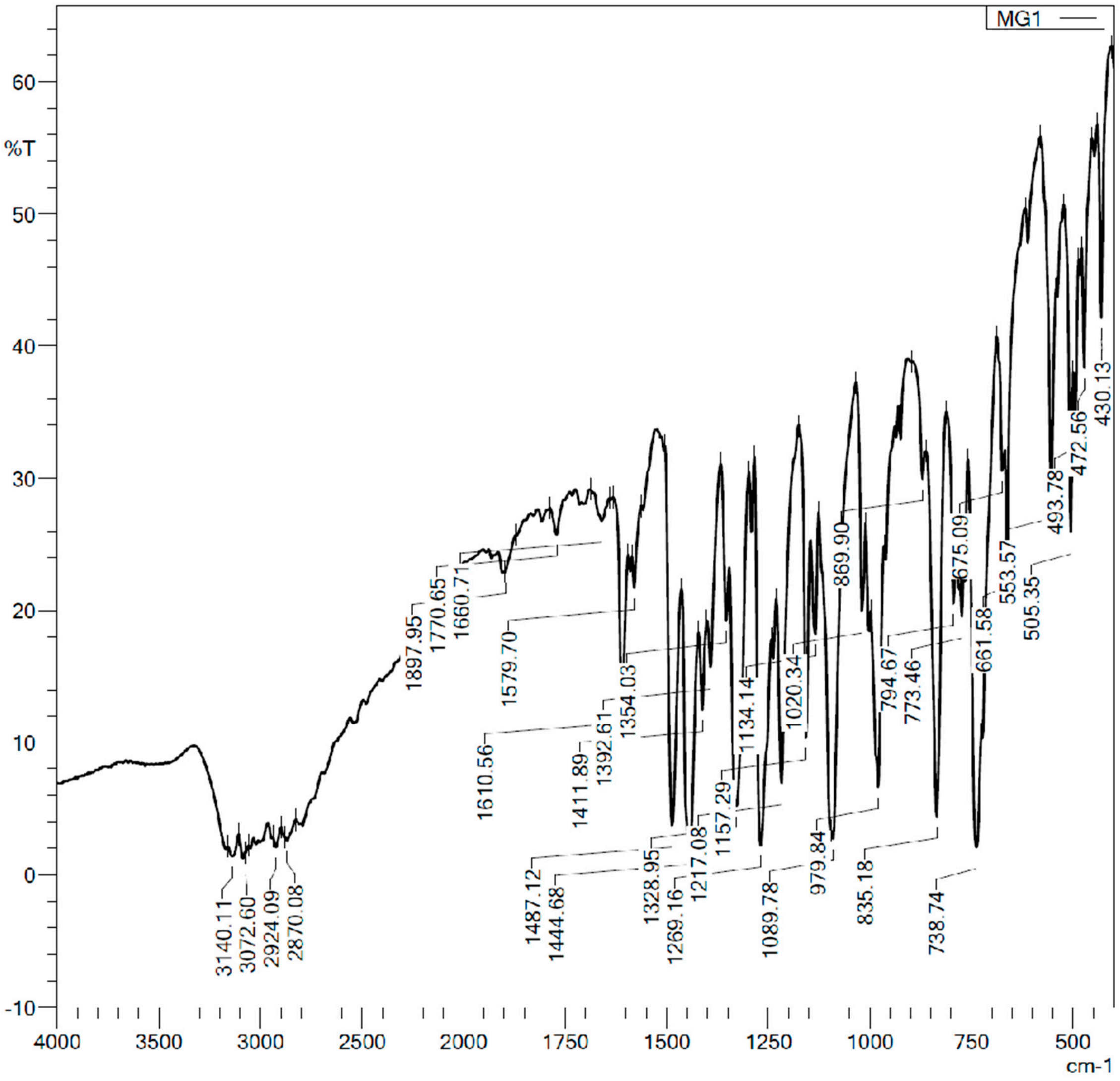


**Figure S3.** FTIR spectra of compound **MG-1**

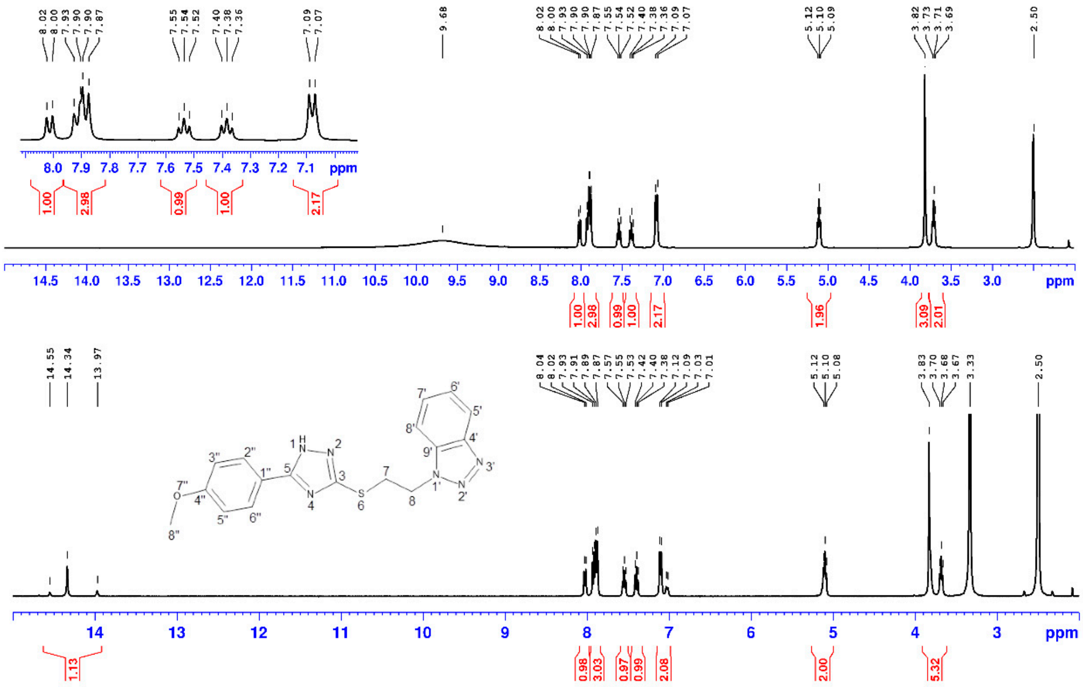

**Figure S4.** $^{1}$H NMR for **MG-2** in DMSO-$d_6$ (bottom) and DMSO-$d_6$ +TFA (up).

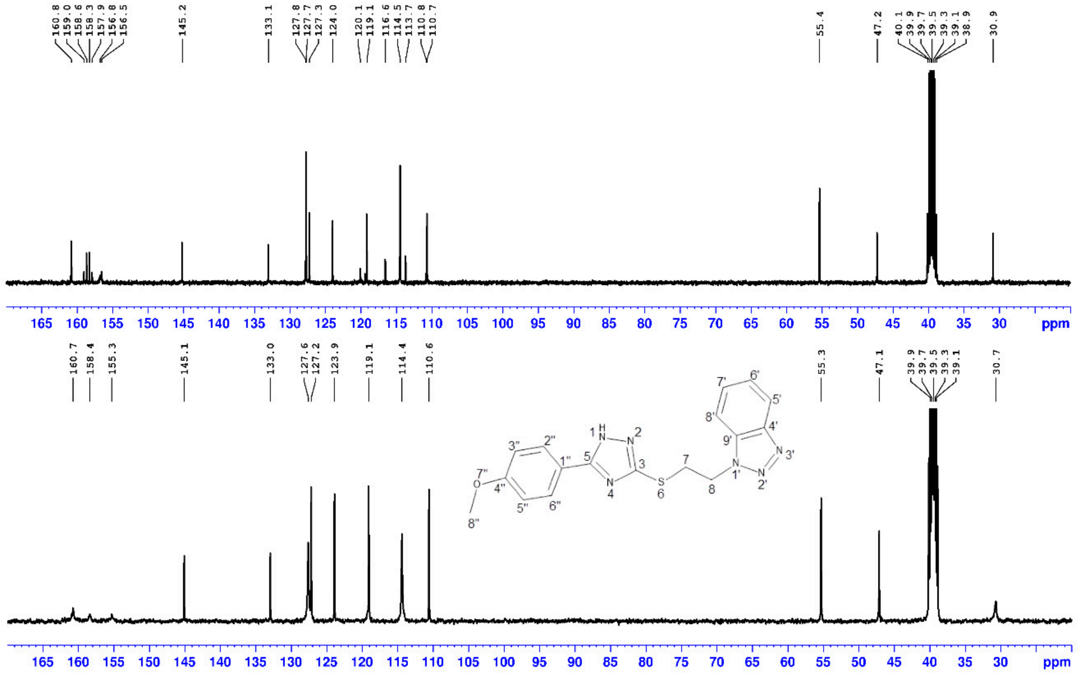

**Figure S5.** $^{13}$C NMR for **MG-2** in DMSO-$d_6$ (bottom) and DMSO-$d_6$ +TFA (up).

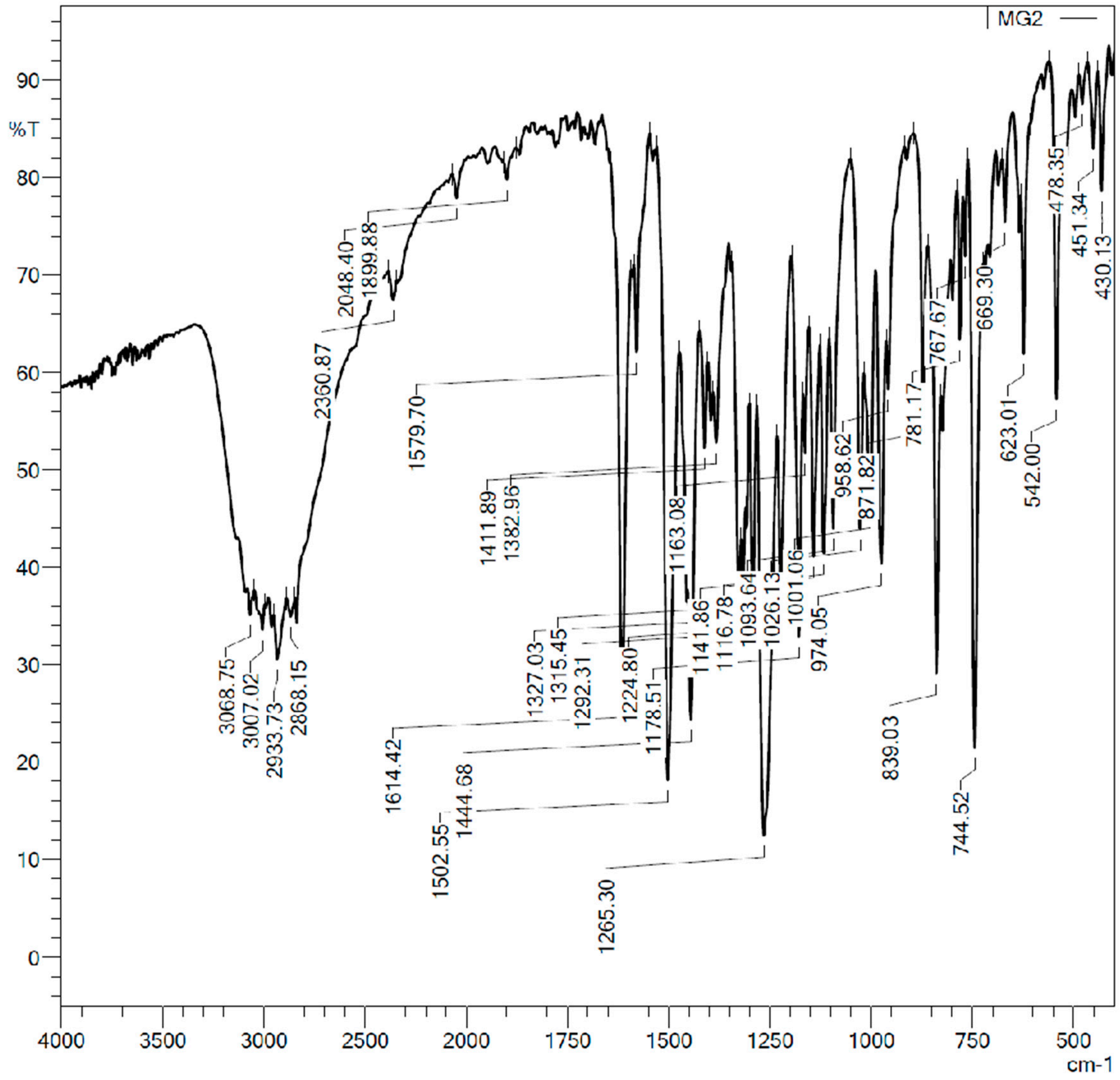


**Figure S6.** FTIR spectra of compound **MG-2**

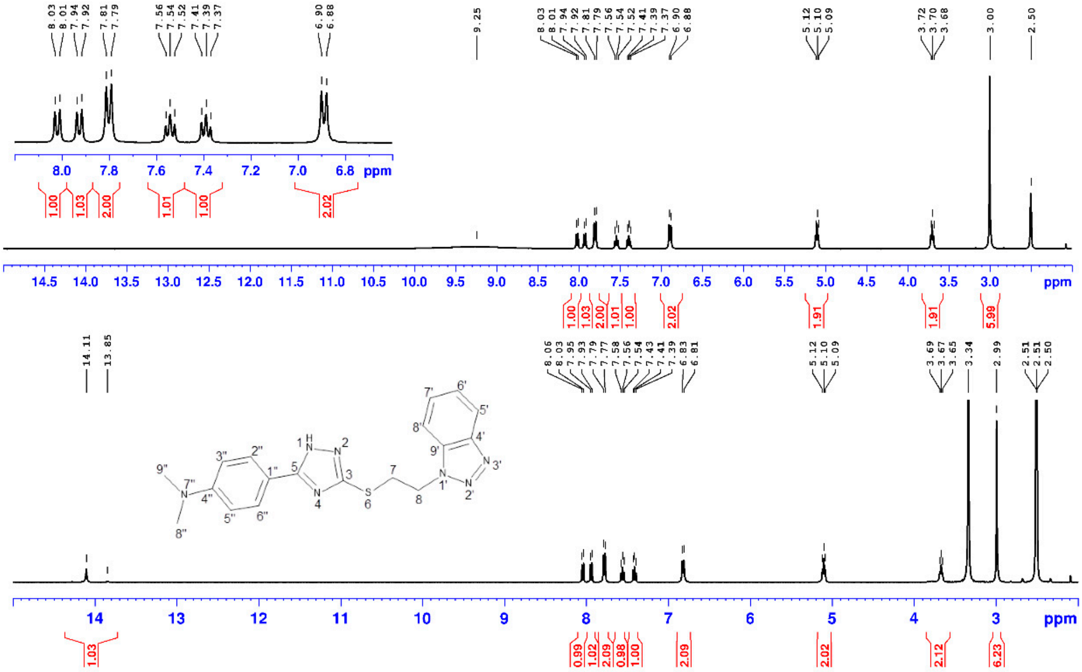


**Figure S7.** $^{1}$H NMR for **MG-3** in DMSO-$d_6$ (bottom) and DMSO-$d_6$ +TFA (up).

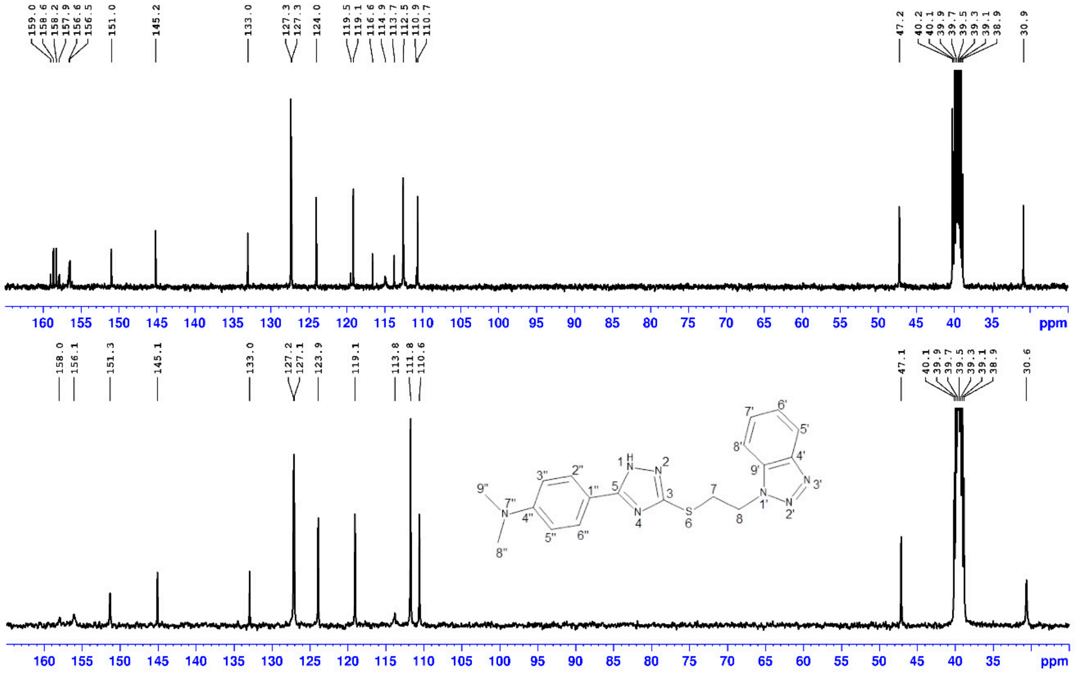


**Figure S8.** $^{13}$C NMR for **MG-3** in DMSO-$d_6$ (bottom) and DMSO-$d_6$ +TFA (up).

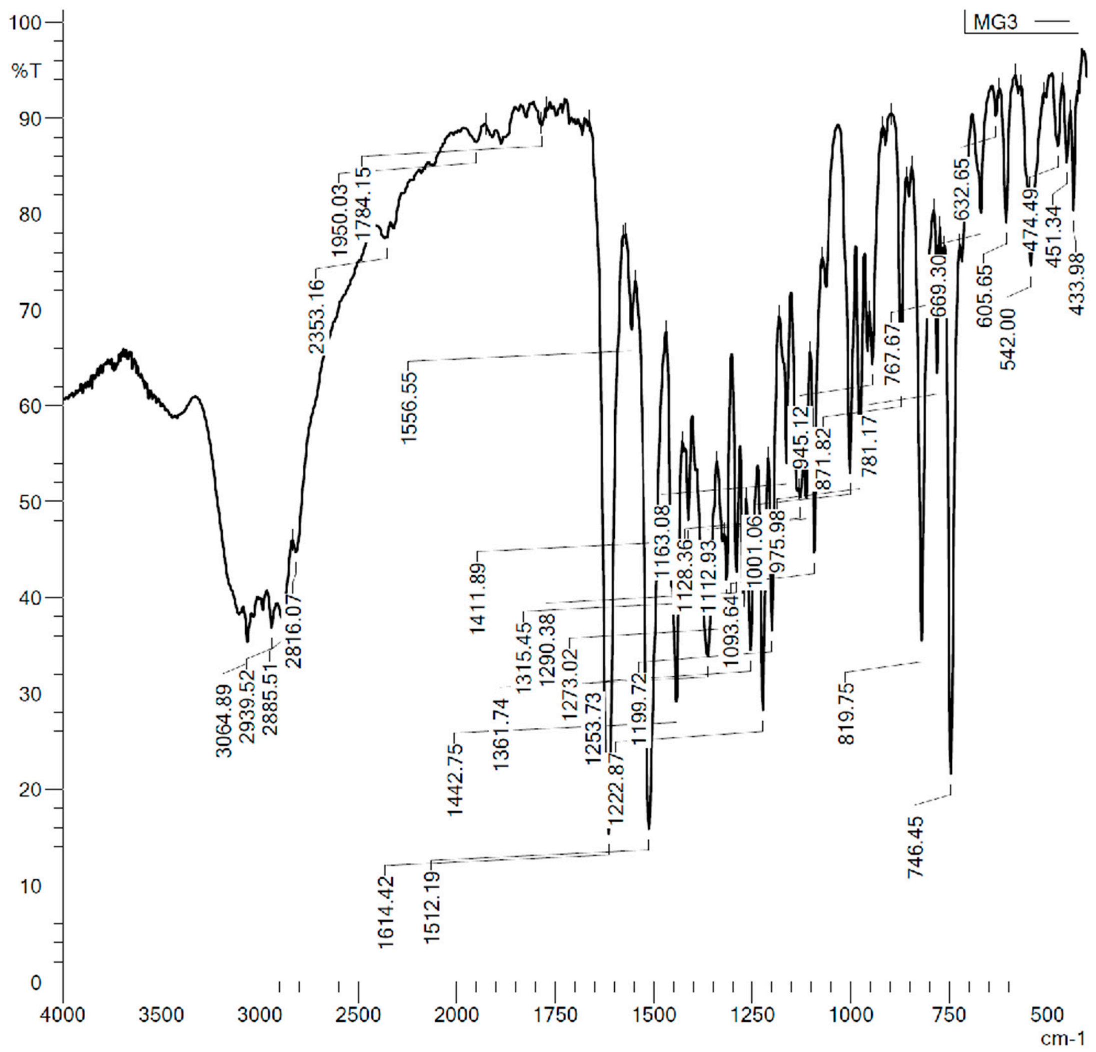


**Figure S9.** FTIR spectra of compound **MG-3**

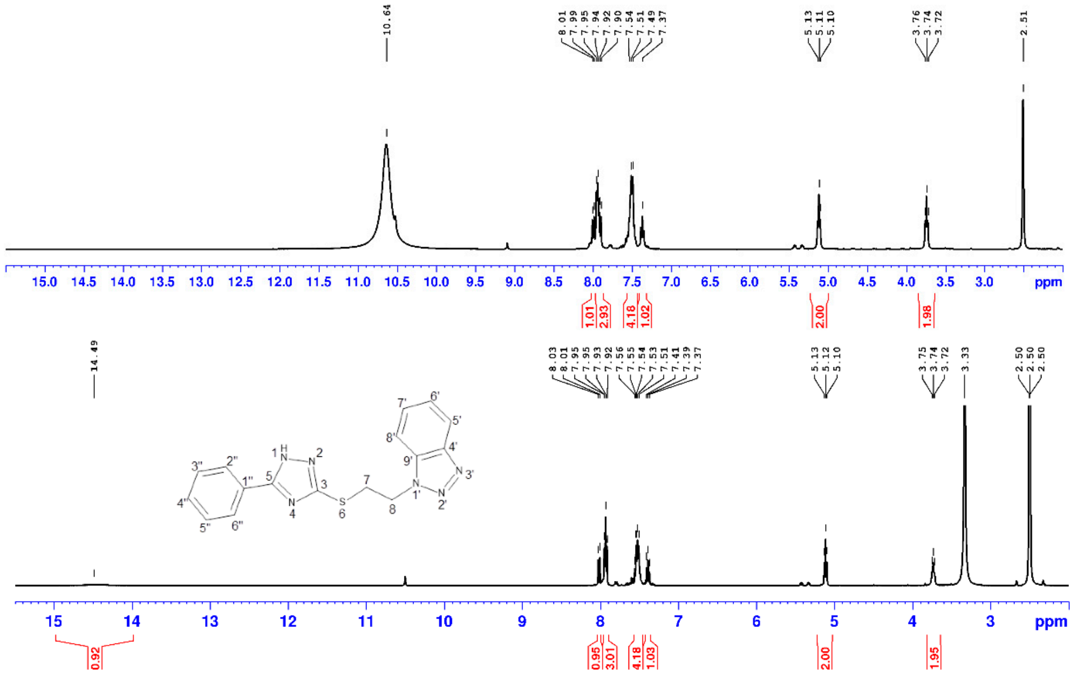


**Figure S10.** $^{1}$H NMR for **MG-4** in DMSO-$d_6$ (bottom) and DMSO-$d_6$ +TFA (up).

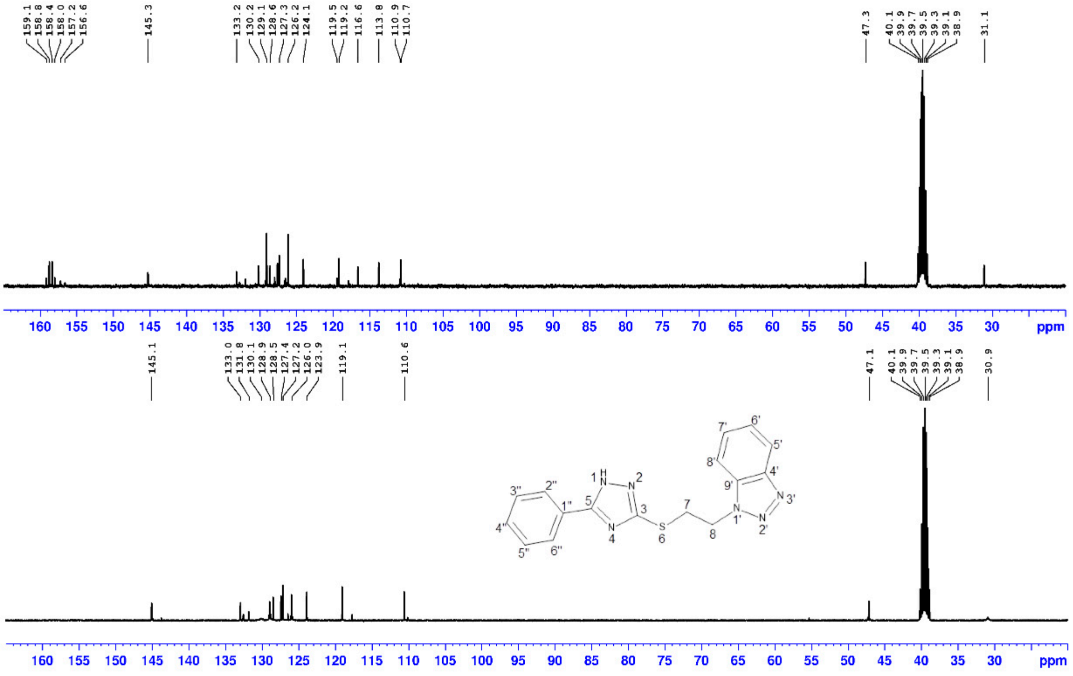


**Figure S11.** $^{13}$C NMR for **MG-4** in DMSO-$d_6$ (bottom) and DMSO-$d_6$ +TFA (up).

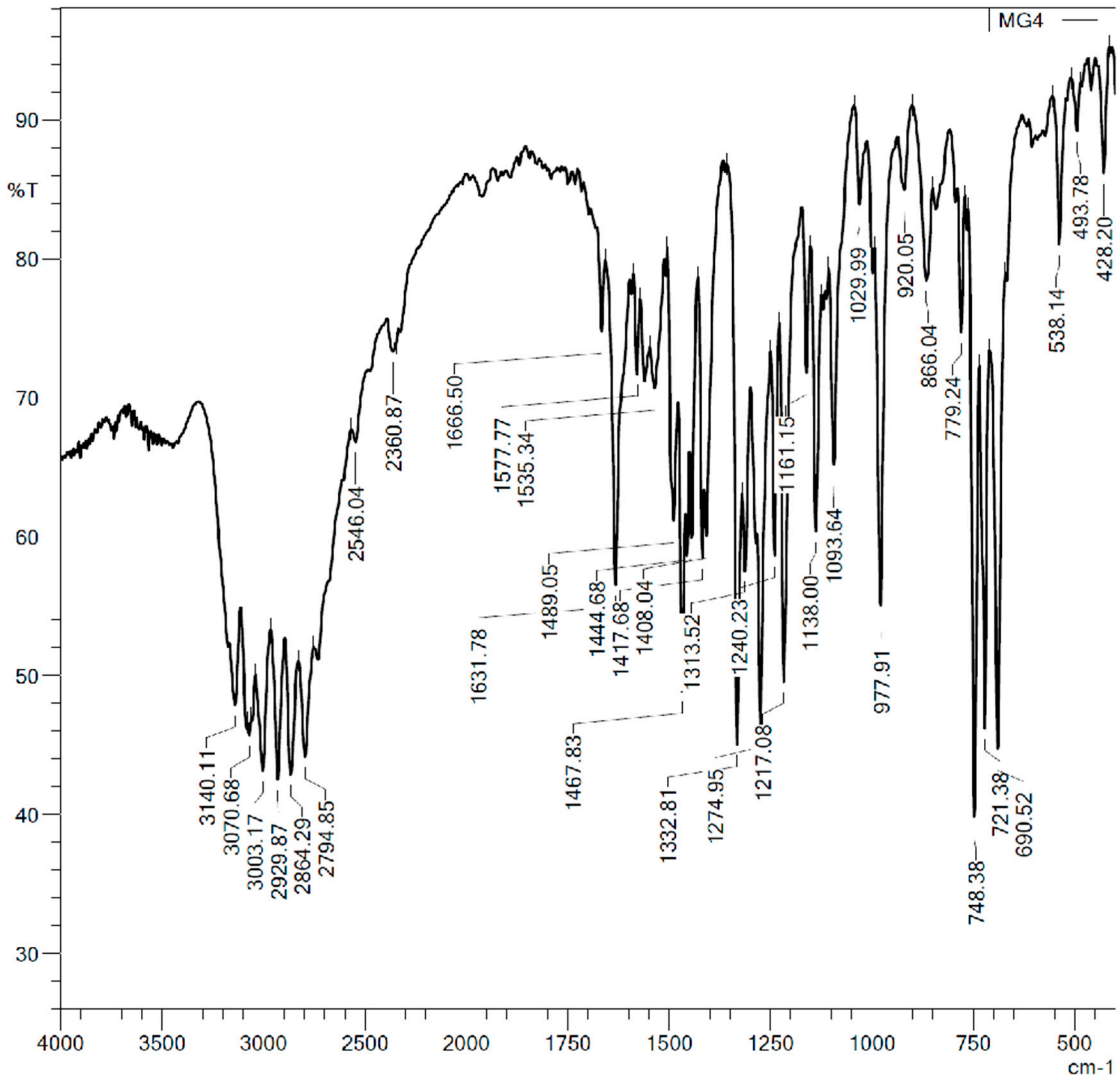


**Figure S12.** FTIR spectra of compound **MG-4**

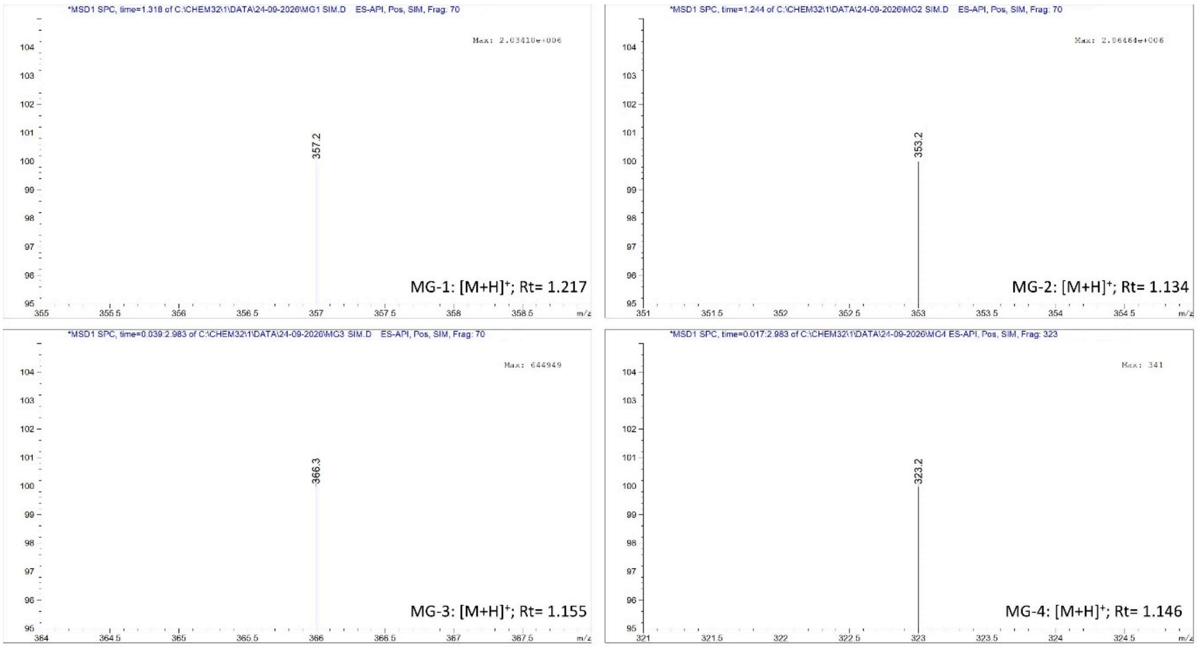


**Figure S13.** LC–MS (ESI$^+$, SIM) spectra of the newly synthesized compounds MG-1–MG-4. The protonated molecular ions $[M+H]^+$ were detected at m/z 357.2 for MG-1 (Rt = 1.217 min), 353.2 for MG-2 (Rt = 1.134 min), 366.3 for MG-3 (Rt = 1.155 min), and 323.2 for MG-4 (Rt = 1.146 min).

## 2. Supplementary cytotoxicity data for synthetic intermediates 1a–1d

### Cell viability results for synthetic intermediates

The cytotoxic effects of the synthetic intermediates (1a, 1b, 1c, and 1d) were evaluated under the same experimental conditions described for the benzotriazole final derivatives in the Materials and Methods section. Thus, the treatment consisted of applying the 4 intermediates to the same 4 cell lines (HaCaT, A375, A549, and HT-29) for 48 h at concentrations of 10, 25, 50, 75, and 100 μM. The results involved assessing cell viability using the Alamar Blue assay as a percentage of the biological control (untreated cells), which is considered 100%. Thus, **Table S1** presents the results for each cell line at a concentration of 100 μM. In the healthy human keratinocyte cell line (HaCaT), the cell viability rate compared to the control group is over 80% for all four compounds. Regarding the cytotoxic effect on the human melanoma cell line (A375), the cell viability rate exceeds 70%, and for the non-small cell lung cancer cell line (A549), it exceeds 75%. A similar result (>75%) was also obtained following the analysis of cell survival in colorectal adenocarcinoma (HT-29). All these results are presented in the four figures (**Figures S14-17**), each representative of a cell line, as well as in **Table S1**. Based on these results obtained with the synthetic intermediates across the four cell lines, only modest reductions in cell viability were observed, with none of the intermediates exhibiting biologically relevant cytotoxic effects within the tested concentration range.

**HaCaT**

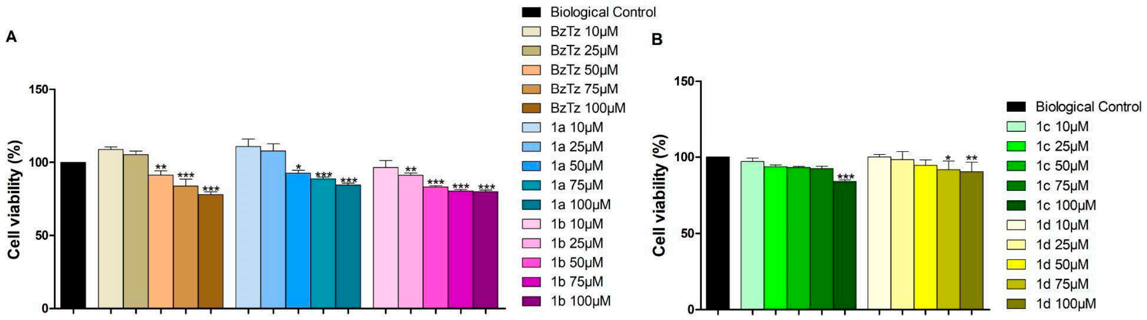


**Figure S14.** Effects of BzTz and synthetic intermediates 1a–1d on HaCaT cell viability after 48 h of treatment. Cell viability was assessed using the Alamar Blue assay and expressed as percentage relative to the biological control (100%). Data represent mean ± SD of three independent experiments performed in triplicate. Differences were considered statistically significant at $p < 0.05$ (* $p < 0.05$, ** $p < 0.01$ and *** $p < 0.001$).

**A375**

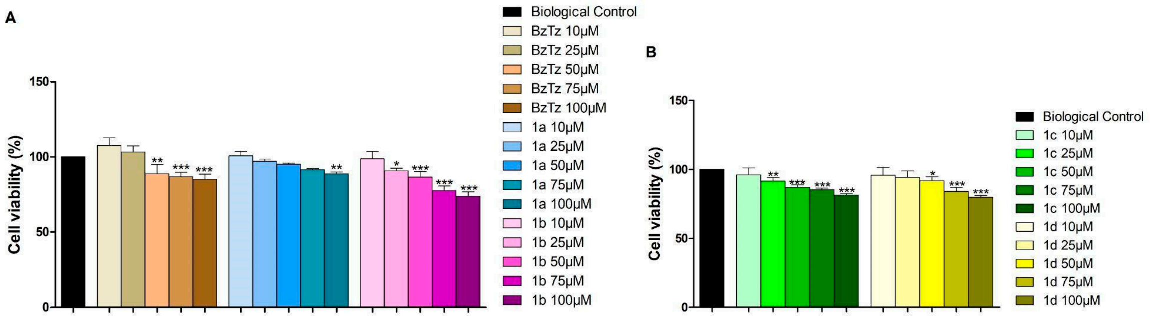


**Figure S15.** Effects of BzTz and synthetic intermediates 1a–1d on A375 cell viability after 48 h of treatment. Cell viability was assessed using the Alamar Blue assay and expressed as percentage relative to the biological control (100%). Data represent mean ± SD of three independent experiments performed in triplicate. Differences were considered statistically significant at $p < 0.05$ (* $p < 0.05$, ** $p < 0.01$ and *** $p < 0.001$).

**A549**

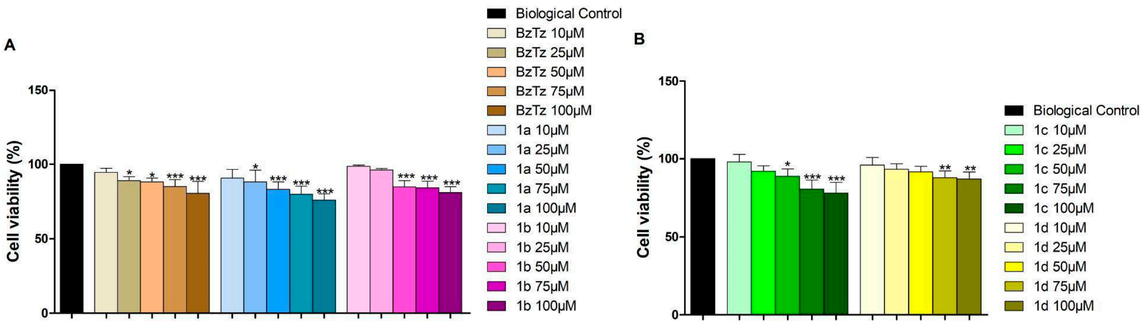


**Figure S16.** Effects of BzTz and synthetic intermediates 1a–1d on A549 cell viability after 48 h of treatment. Cell viability was assessed using the Alamar Blue assay and expressed as percentage relative to the biological control (100%). Data represent mean ± SD of three independent experiments performed in triplicate. Differences were considered statistically significant at $p < 0.05$ (* $p < 0.05$, ** $p < 0.01$ and *** $p < 0.001$).

**HT-29**

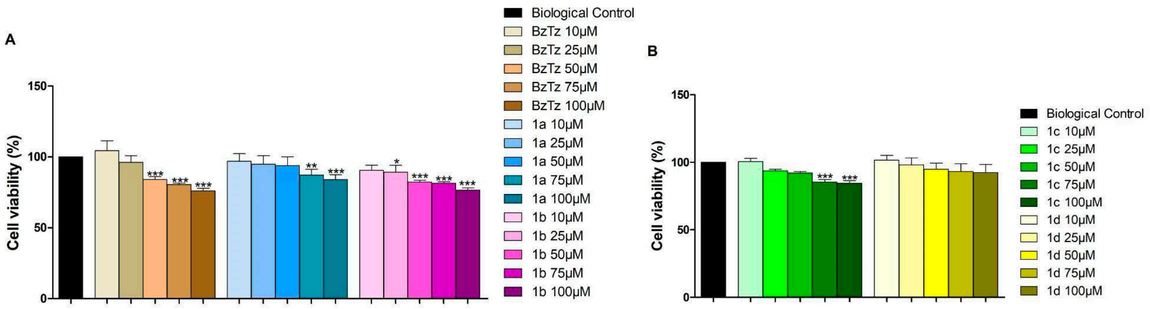


**Figure S17.** Effects of BzTz and synthetic intermediates 1a–1d on HT-29 cell viability after 48 h of treatment. Cell viability was assessed using the Alamar Blue assay and expressed as percentage relative to the biological control (100%). Data represent mean ± SD of three independent experiments performed in triplicate. Differences were considered statistically significant at $p < 0.05$ (* $p < 0.05$, ** $p < 0.01$ and *** $p < 0.001$).

**Table S1. Summary of cell viability values at 100 µM for BzTz and synthetic intermediates 1a–1d.**

This table summarizes the highest-concentration screening data for the synthetic intermediates across the four tested cell lines. Values are expressed as percentage of the biological control and reported as mean ± SD after 48 h of treatment.

| Cell line | BzTz | 1a | 1b | 1c | 1d |
|---|---|---|---|---|---|
| **HaCaT** | 78.06% ± 1.9 | 84.59% ±1.22 | 80.09% ±0.92 | 84.03% ±1.11 | 90.44% ±6.14 |

| | | | | | |
|---|---|---|---|---|---|
| **A375** | 85.21% ± 3.35 | 88.78% ±1.30 | 73.83% ±2.96 | 81.38% ±0.97 | 79.70% ±1.29 |
| **A549** | 80.47% ± 8.02 | 75.92% ±4.29 | 80.91% ±4.05 | 78.06% ±6.65 | 86.87% ±4.59 |
| **HT-29** | 76.08% ± 1.85 | 83.94% ±3.67 | 76.62% ±1.58 | 84.63% ±1.71 | 92.52% ±5.91 |

## 3. Supplementary antimicrobial evaluation of BzTz, synthetic intermediates 1a–1d, and final benzotriazole–1,2,4-triazole hybrids MG-1–MG-4

### Antimicrobial activity assay

The diffusion method [1] was used to analyze the antimicrobial activity of BzTz, its final derivatives (MG-1–MG-4), and its intermediate derivatives (1a–d). The microorganisms tested included Gram-positive bacteria (Staphylococcus aureus, Streptococcus pyogenes, and Streptococcus pneumoniae), Gram-negative bacteria (Escherichia coli), and two fungal strains (Candida albicans and Candida parapsilosis) can be found in **Table S2.**

**Table S2.** Microbial species tested

| **Microbial strains** | **ATCC** |
|---|---|
| *Staphylococcus aureus* | 29213 |
| *Streptococcus pyogenes* | 19615 |
| *Streptococcus pneumoniae* | 49619 |
| *Escherichia coli* | 25922 |
| *Candida albicans* | 10231 |
| *Candida parapsilosis* | 22019 |

For testing each strain included in the study **(Table S2),** standardized suspensions in physiological saline at a turbidity of 0.5 McFarland were used. Thus, Mueller–Hinton agar was used for the inoculation of *Staphylococcus aureus* and *Escherichia coli*. In contrast, Mueller–Hinton agar supplemented with defibrinated horse blood and β-NAD was used for the inoculation of *Streptococcus pyogenes* and *Streptococcus pneumoniae*. For the two fungal strains (*Candida spp.*), Mueller–Hinton agar + 2% glucose + methylene blue was used for inoculation. Each chemical compound (BzTz, intermediate derivatives, and final derivatives) was dissolved in DMSO and tested at 500 μg/mL. Thus, a volume of 15 μL of each test solution was applied to sterile 7-mm white paper discs, which were then placed on the inoculated agar surface. DMSO was tested as a negative control, while levofloxacin and fluconazole were used as positive controls for bacterial and fungal strains, respectively. After treatment, the plates

were incubated at 35°C for 24 hours, after which the inhibition zones were measured in millimeters. Since no detectable inhibition was observed in tests conducted at a concentration of 500 μg/mL, it was not necessary to evaluate efficacy at lower concentrations. Thus, all compounds that exhibited only a disc diameter, without a clear zone of inhibition—with the exception of the controls—were reported as inactive.

**Antimicrobial activity results**

The antimicrobial effects of BzTz, the synthetic intermediates 1a–1d, and its final derivatives (MG-1-MG-4) were evaluated at the concentration (500 μg/mL) against six strains, including: *Staphylococcus aureus, Streptococcus pyogenes, Streptococcus pneumoniae, Escherichia coli, Candida albicans,* and *Candida parapsilosis*. The results of the treatment revealed that none of the tested compounds (BzTz, 1a-d, and MG-1-MG-4) exhibited any detectable antimicrobial effect, as the diameters of the treated zones were similar to both the disc diameter and those of the negative control, DMSO; suggesting the absence of inhibition zones. On the other hand, positive controls—specifically levofloxacin for bacterial strains [2] and fluconazole for fungal strains [3]—generated inhibition zones directly proportional to antimicrobial sensitivity, validating the analysis. These results indicate that the compounds under study, under the current experimental conditions, do not exhibit antimicrobial activity (antibacterial and antifungal). All the data can be found in **Table S3.**

**Table S3. Antimicrobial activity of BzTz and synthetic intermediates 1a–1d, and final benzotriazole–1,2,4-triazole hybrids MG-1–MG-4 at 500 μg/mL**

| **Tested condition** | ***Staphylococcus aureus*** **ATCC 29213** | ***Streptococcus pyogenes*** **ATCC 19615** | ***Streptococcus pneumoniae*** **ATCC 49619** | ***Escherichia coli*** **ATCC 25922** | ***Candida albicans*** **ATCC 10231** | ***Candida parapsilosis*** **ATCC 22019** |
|---|---|---|---|---|---|---|
| **Positive control** | Levofloxacin 23 mm | Levofloxacin 20 mm | Levofloxacin 27 mm | Levofloxacin 22 mm | Fluconazole 21 mm | Fluconazole 33 mm |
| **DMSO** | 7 mm / NA | 7 mm / NA | 7 mm / NA | 7 mm / NA | 7 mm / NA | 7 mm / NA |
| **BzTz** | 7 mm / NA | 7 mm / NA | 7 mm / NA | 7 mm / NA | 7 mm / NA | 7 mm / NA |
| **1a** | 7 mm / NA | 7 mm / NA | 7 mm / NA | 7 mm / NA | 7 mm / NA | 7 mm / NA |
| **1b** | 7 mm / NA | 7 mm / NA | 7 mm / NA | 7 mm / NA | 7 mm / NA | 7 mm / NA |

| | | | | | | |
|---|---|---|---|---|---|---|
| **1c** | 7 mm / NA | 7 mm / NA | 7 mm / NA | 7 mm / NA | 7 mm / NA | 7 mm / NA |
| **1d** | 7 mm / NA | 7 mm / NA | 7 mm / NA | 7 mm / NA | 7 mm / NA | 7 mm / NA |
| **MG-1** | 7 mm / NA | 7 mm / NA | 7 mm / NA | 7 mm / NA | 7 mm / NA | 7 mm / NA |
| **MG-2** | 7 mm / NA | 7 mm / NA | 7 mm / NA | 7 mm / NA | 7 mm / NA | 7 mm / NA |
| **MG-3** | 7 mm / NA | 7 mm / NA | 7 mm / NA | 7 mm / NA | 7 mm / NA | 7 mm / NA |
| **MG-4** | 7 mm / NA | 7 mm / NA | 7 mm / NA | 7 mm / NA | 7 mm / NA | 7 mm / NA |

**Note.** Data are expressed as inhibition zone diameters in millimeters. A value of 7 mm corresponds to the paper disk diameter and indicates absence of detectable antimicrobial activity. NA, no antimicrobial activity detected under the tested conditions. Levofloxacin was used for bacterial strains; fluconazole was used for *Candida spp.*

**Interpretation and limitations of the antimicrobial screening**

The results of the antimicrobial activity study showed a completely negative result at a concentration of 500 µg/mL for both the MG-1–MG-4 series and the 1a–d series. In contrast, the results were in accordance with the guidelines for both positive controls used to measure the antibacterial (levofloxacin [2]) and antifungal (fluconazole [3]) effects, under the same experimental conditions and on the same 6 strains (*Staphylococcus aureus, Streptococcus pyogenes, Streptococcus pneumoniae, Escherichia coli, Candida albicans,* and *Candida parapsilosis*). Thus, it was not necessary to test concentrations lower than 500 µg/mL or to determine minimum inhibitory concentrations in the disk diffusion screening test used in this study. Furthermore, the results may be limited to the absence of detectable antimicrobial activity, without generalizing the inactivity of these compounds.

## References


1. European Committee on Antimicrobial Susceptibility Testing (EUCAST) *EUCAST Disk Diffusion Method for Antimicrobial Susceptibility Testing*; 2025;
2. Pfaller, M.A.; Barry, A.L.; Fuchs, P.C. Levofloxacin Disk Potency and Tentative Interpretive Criteria for Susceptibility Tests. *J. Clin. Microbiol.* **1993**, *31*, 1924–1926, doi:10.1128/jcm.31.7.1924-1926.1993.
3. Pfaller, M.A.; Diekema, D.J.; Sheehan, D.J. Interpretive Breakpoints for Fluconazole and *Candida* Revisited: A Blueprint for the Future of Antifungal Susceptibility Testing. *Clin. Microbiol. Rev.* **2006**, *19*, 435–447, doi:10.1128/CMR.19.2.435-447.2006.
